# Nonsense mutation in *PMEL* is associated with yellowish plumage colour phenotype in Japanese quail

**DOI:** 10.1038/s41598-018-34827-4

**Published:** 2018-11-13

**Authors:** Satoshi Ishishita, Mayuko Takahashi, Katsushi Yamaguchi, Keiji Kinoshita, Mikiharu Nakano, Mitsuo Nunome, Shumpei Kitahara, Shoji Tatsumoto, Yasuhiro Go, Shuji Shigenobu, Yoichi Matsuda

**Affiliations:** 1 Avian Bioscience Research Center, Graduate School of Bioagricultural Sciences, Nagoya University, Furo-cho, Chikusa-ku, Nagoya, Aichi 464-8601 Japan; 2 Laboratory of Animal Genetics, Department of Applied Molecular Biosciences, Graduate School of Bioagricultural Sciences, Nagoya University, Furo-cho, Chikusa-ku, Nagoya, Aichi 464-8601 Japan; 3 Functional Genomics Facility, National Institute for Basic Biology, Okazaki, Aichi 444-8585 Japan; 4 Exploratory Research Center on Life and Living Systems (ExCELLS), National Institutes of Natural Sciences, Okazaki, Aichi 444-8585 Japan; 5 National Institute for Physiological Sciences, Okazaki, Aichi 444-8585 Japan

## Abstract

The L strain of Japanese quail exhibits a plumage phenotype that is light yellowish in colour. In this study, we identified a nonsense mutation in the *premelanosome protein* (*PMEL*) gene showing complete concordance with the yellowish plumage within a pedigree as well as across strains by genetic linkage analysis of an F_2_ intercross population using approximately 2,000 single nucleotide polymorphisms (SNPs) that were detected by double digest restriction site-associated DNA sequencing (ddRAD-seq). The yellowish plumage was inherited in an autosomal recessive manner, and the causative mutation was located within an 810-kb genomic region of the LGE22C19W28_E50C23 linkage group (LGE22). This region contained the *PMEL* gene that is required for the normal melanosome morphogenesis and eumelanin deposition. A nonsense mutation that leads to a marked truncation of the deduced protein was found in *PMEL* of the mutant. The gene expression level of *PMEL* decreased substantially in the mutant. Genotypes at the site of the nonsense mutation were fully concordant with plumage colour phenotypes in 196 F_2_ offspring. The nonsense mutation was not found in several quail strains with non-yellowish plumage. Thus, the yellowish plumage may be caused by the reduced eumelanin content in feathers because of the loss of PMEL function.

## Introduction

Melanin pigments in the skin, hair, and eyes have many biological functions, such as absorption of ultraviolet light, scavenging free radicals, concealing and warning colouration, and sexual communication^1^. They are also involved in the development of the optic nervous system and in retinal function^2,3^. Melanin typically consists of two types of molecules: black or brown eumelanin, and yellow or red pheomelanin^4^. These are synthesised within a lysosome-related organelle called a melanosome, which functions in the protection of cytosolic components from oxidative attack during melanin synthesis and is also involved in the storage and transfer of melanin^5^. Melanosomes that generate predominantly eumelanin mature through four morphologically distinct stages of development^5–7^. They first appear as vacuolar endosomes (stage I) and then acquire intraluminal proteinaceous fibrils (stages I and II). The first two stages of melanosomes lack pigments. Melanin begins to be deposited onto fibrillar matrix (stage III), and eventually melanin-dense mature melanosomes are formed (stage IV). Many causative genes of plumage colour mutants have been identified in birds, including chickens, Japanese quail, and doves^8–21^, and the mutant animals have contributed to the investigation of *in vivo* functions of these genes involved in the biosynthesis of melanin.

**Figure 1:**
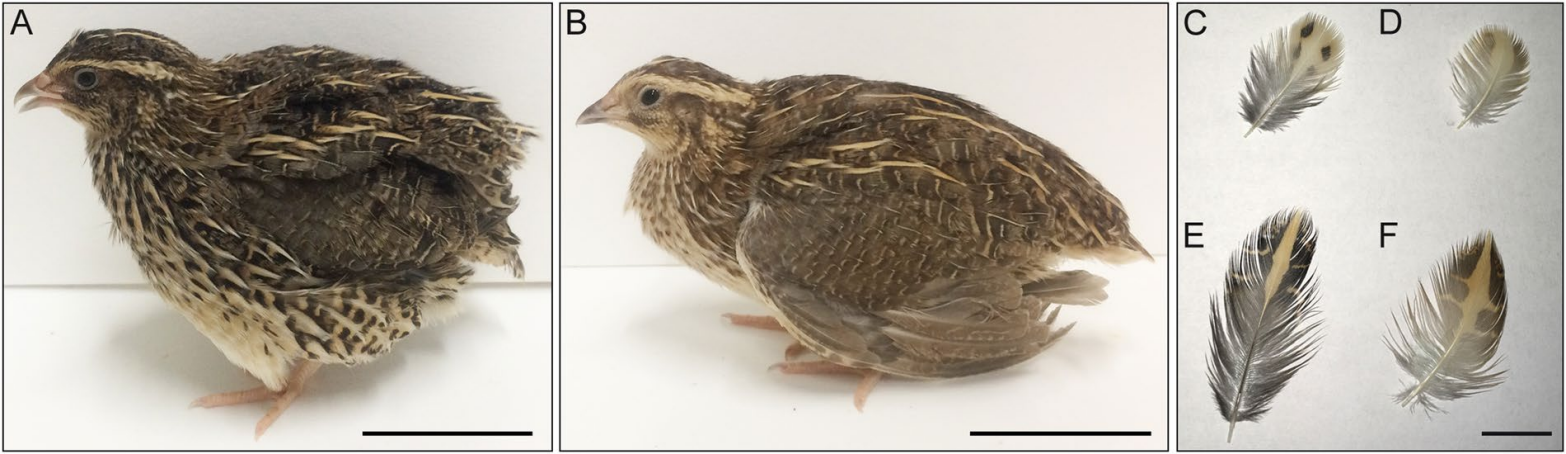
Plumage colour phenotypes of the wild-type and mutant quail. A female of the WE strain exhibiting the wild-type plumage colour (**A**) and a female of the L strain exhibiting yellowish mutant plumage colour (**B**). Feathers around the chest (**C**,**D**) and the back (**E**,**F**). Colour patterns in feathers, such as spots and stripes, are unclear in the L strain (**D**–**F**), compared with the WE strain (**C**–**E**). Dark-coloured regions were observed in feathers of both strains; however, feather colour of the L strain was light and yellowish, compared with the WE strain. Scale bars, 50 mm in the left and middle panels, 10 mm in the right panel.

The L strain of Japanese quail (*Coturnix japonica*; hereafter referred to as quail) has been established by the selective breeding of animals with low antibody production against an inactivated Newcastle disease virus (NDV) antigen. It has been maintained for more than 50 generations at the National Institute for Environmental Studies (NIES), Japan^22,23^: after the first 35 generations of breeding based on their anti-inactivated NDV antibody titres, quail have been maintained by pair-mating in closed colonies. The L strain is also characterised by yellowish plumage colour (Fig. 1) in both sexes. This plumage colour phenotype is only known in quail in the L strain. No abnormal phenotypes other than plumage colour are known in this strain. Neither the mode of inheritance, nor the causative gene of the yellowish plumage, have been identified.

In this study, to identify the causative gene for yellowish plumage in the L strain, we performed genetic linkage analysis using F_2_ progeny between the L strain and the WE strain exhibiting wild-type plumage colour^24^, with many single-nucleotide polymorphisms (SNPs) detected by double digest restriction site-associated DNA sequencing (ddRAD-seq)^25^. Then, we searched for the candidate mutation in the causative genomic region.

## Results

### Yellowish plumage is inherited in an autosomal recessive manner

We initially generated F_2_ offspring from F_1_ hybrids between a single female of the L strain and a single male of the WE strain. The L and WE strains exhibit yellowish plumage and wild-type plumage, respectively (Fig. 1). A total of 378 F_2_ individuals were generated by crossing a single F_1_ male with three F_1_ females. Yellowish plumage was observed in 83 offspring and wild-type plumage was observed in 295 offspring. The segregation ratio of phenotypes conforms to autosomal recessive inheritance (Chi-squired test, P > 0.05), which indicates that the yellowish plumage is controlled by a single autosomal recessive gene. We named the causative mutation for this plumage colour phenotype *yellowish* (*yw*).

### The *yw* locus was mapped to an 810-kb genomic region on LGE22

DNA samples of 96 male and 100 female F_2_ offspring were used for ddRAD-seq. We prepared three ddRAD-seq libraries and conducted a single run of DNA sequencing per library (see Methods for details). Identification of informative SNP markers (hereafter also referred to as markers) and subsequent genotyping of the F_2_ offspring were performed using the Stacks program^26^. Loci with a low coverage depth (less than eight reads) in each individual were set as missing values, and markers which could not be assigned to known chromosomes or linkage groups were eliminated. To select markers for a case-control association test, we constructed a genetic linkage map (hereafter referred to as genetic map) with the genotype data of 89 F_2_ males and 92 F_2_ females using the Lep-MAP2 program (LM2)^27^ after pre-mapping quality control. The pre-mapping quality control eliminated markers and individuals with a high missing genotype rate and segregation distortion (see Methods for details). The average coverage depth of each sample is shown in Fig. S1. After correction of erroneous marker ordering that was output by LM2, we eventually constructed a genetic map that consisted of 2,004 markers that included 1,949 autosomal markers and 55 Z-linked markers (Fig. S2). The total map distance was approximately 2,350 cM, and the average intermarker distance was 1.2 cM (Table S1, Fig. S3). Recoquillay and colleagues^28^ previously reported a genetic map of quail with a total distance of 3,057 cM. This genetic map was constructed with approximately 1,500 SNP markers obtained by whole genome sequencing of F_2_ samples using the chicken genome assembly as a reference. The markers used in our genetic map covered 95% of the genomic region in terms of physical length; however, markers were missing from substantial parts of several chromosomes (Table S1), which may be the main reason for the difference in the total distance of the genetic map between the previous study and the present study. It should be noted that the small number of markers on chromosome 16 is due to the presence of MHC gene clusters on this chromosome^29^. Markers that were mapped to LGE22 and LGE64 showed no linkage to any markers on chromosomes in the genome assembly.

**Figure 2:**
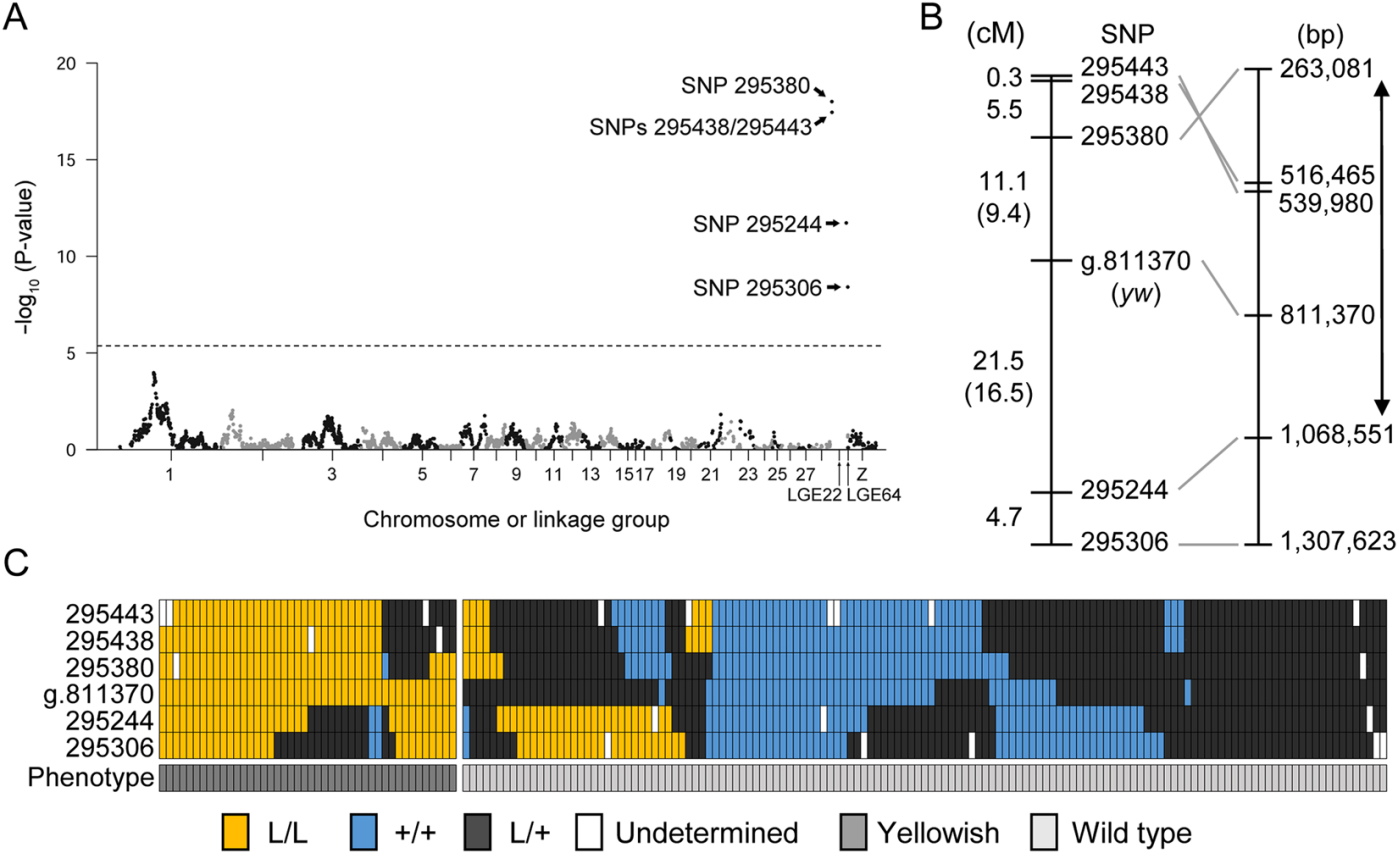
Case-control association test and genetic mapping. (**A**) Manhattan plot showing the association between SNP markers on LGE22 and plumage phenotypes. The horizontal axis shows map positions (cM) of SNP markers on each chromosome or linkage group, and the vertical axis shows the negative logarithm of the unadjusted *P*-value for each SNP marker. The dashed line shows the level of Bonferroni-corrected 1% significance. The top three SNP *P*-values are 5.1 × 10^−22^ in SNP 295380, 9.8 × 10^−29^ in SNP 295438, and 3.5 × 10^−18^ in SNP 295443. (**B**) Locations of SNP markers and the *yw* locus on the genetic map. Names of SNP markers are shown in the middle; genetic distances between these SNP markers are shown in the left; nucleotide positions of these SNP markers in the NC_029544.1 reference genome assembly (*Coturnix japonica* 2.0) are shown in the right. Genetic distances between the *yw* locus and its flanking markers, which were calculated only using F_2_ individuals exhibiting yellowish plumage, are shown in parentheses. Two direction-arrow indicates the causative region. The SNP at the site of the nonsense mutation is indicated by ‘g.811370’. It should be noted that the order of SNPs 295380, 295438, and 295443 was inverted between the genetic map and the reference genome assembly. (**C**) Genotypes and phenotypes of 181 F_2_ offspring. Rectangles indicate genotypes of SNP markers and phenotypes of F_2_ individuals. Yellow, homozygous mutant genotype (L/L); blue, wild-type genotype (+/+); dark grey, heterozygous genotype (L/+); white, undetermined genotype; grey, yellowish plumage; light grey, wild-type plumage. The genotype and phenotype of each F_2_ individual is represented as a column of rectangles.

Subsequently, a case-control association test was performed using markers in the genetic map. The result showed that yellowish plumage was highly associated with markers located on LGE22 (Fig. 2A). Then, we estimated recombination fractions and LOD (logarithm of the odds ratio) scores between the *yw* locus and all markers on LGE22. The result showed that the *yw* locus was located within an interval between SNPs 295380 and 295244, whose positions in the reference genome assembly were 263 and 1,068 kb, respectively (Fig. 2B,C, Table S2). The physical distance between these two markers was 810 kb; however, we found that the order of SNPs 295443, 295438, and 295380 in the reference genome assembly was the opposite of that in the genetic map (Fig. 2B). This may be due to an error in the genomic sequence of LGE22. An alternative possibility is the structural variation of chromosomes between the quail used for the draft genome assembly and those used in the present study. A chromosomal inversion between SNPs 295443 and 295380, or a translocation of the genomic region containing SNP 295380 or SNPs 295443 and 295438 may have occurred. We assumed that the 810-kb genomic region is the causative genomic region. The causative region includes a total of 67 genes, which consist of 64 protein-coding genes and three non-coding genes (*LOC107325707, LOC107325749*, and *TRNAS-CGA*) (Table S3). When we searched for the functions of their human homologs using UniProt Knowledgebase^30^, only *PMEL* was found to be related to melanin biosynthesis. PMEL functions specifically in pigment cells and is required for the normal melanosome formation and eumelanin production, which is evolutionarily conserved in vertebrates^31–33^. Thus, we next searched for a mutation in the *PMEL* coding sequence.

### A nonsense mutation in *PMEL* was found in the L strain genome, but not in other quail strain’s genomes

**Figure 3:**
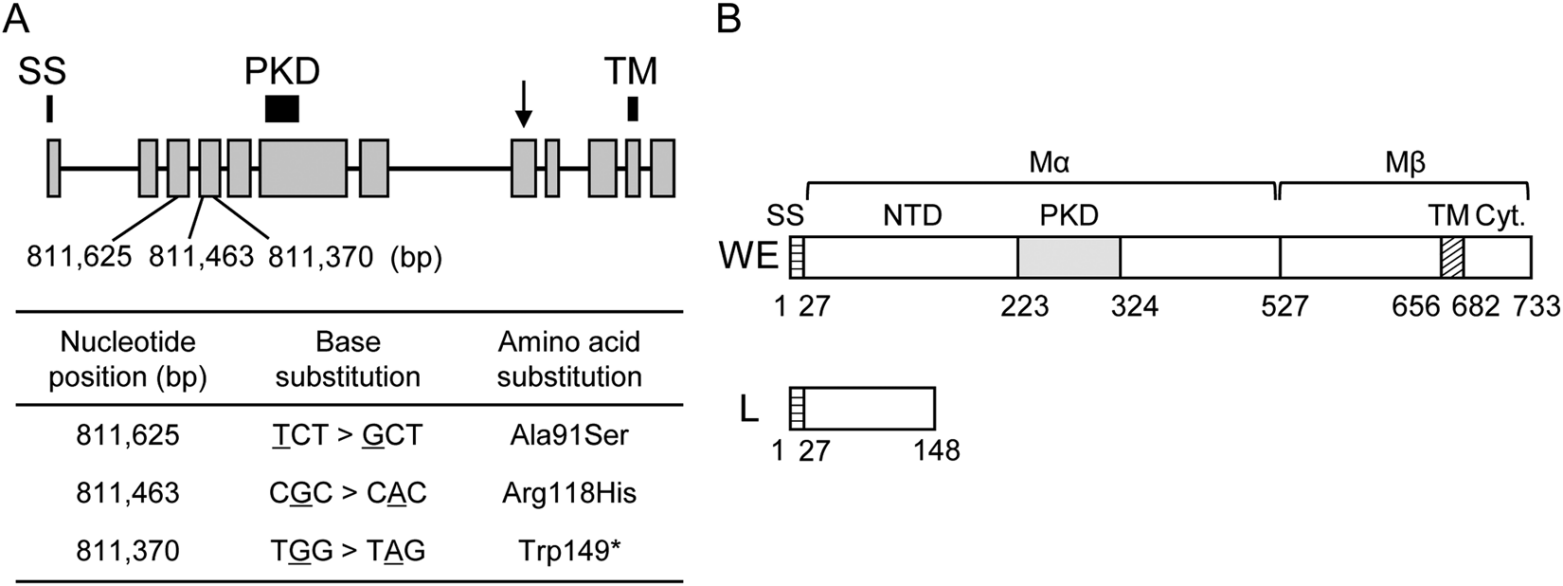
Genomic positions of mutations in the *PMEL* gene and structures of wild-type and mutant-type PMEL proteins. (**A**) Schematic representation of the *PMEL* gene and nucleotide positions of mutations in LGE22. Grey boxes and black lines indicate exons and introns, respectively. Black bars indicate signal sequence (SS), polycystic kidney disease (PKD), and transmembrane (TM) domains. The arrow indicates the proteolytic cleavage site. Nucleotide positions (bp) of mutations in LGE22 in the quail genome assembly are indicated at the bottom of the diagram. The table indicates base substitutions and amino-acid substitutions due to these mutations. A nonsense mutation was found at the nucleotide position 811,370 in the fourth exon of the *PMEL* gene. Two missense mutations were found upstream of the nonsense mutation. (**B**) Schematic representation of deduced PMEL proteins of the WE and L strains. The PMEL of the WE strain contains SS, the amino-terminal domain (NTD), and PKD, TM, and cytoplasmic (Cyt.) domains. SS is removed cotranslationally, and Mα and Mβ fragments are generated by proteolytic cleavage in the Golgi apparatus or a post Golgi compartment^31^. The PMEL of the L strain contains SS and part of the NTD.

Sequencing of *PMEL* cDNA, which was synthesized using total RNA isolated from 11-day-old whole embryos of the L and WE strains, revealed the presence of multiple base substitutions and a single 24-bp insertion in the coding sequence (CDS) (Figs S4 and S5). Of these polymorphisms, a G-A base substitution at the 446th nucleotide of the CDS leads to the creation of a stop codon, which causes premature termination of *PMEL* in the L strain (Figs 3A and S6). The nonsense mutation was mapped within the 4th exon of the *PMEL* gene (Fig. 3A). Two missense mutations were found upstream of the nonsense mutation (Figs 3A and S6). The CDS of a wild-type *PMEL* is 2,202-bp long and encodes a protein of 733 amino acids; however, the deduced PMEL protein of the L strain was 148-amino acids long, containing only a signal sequence and part of the N-terminal domain (NTD) (Figs 3B and S7). The site of the nonsense mutation was located at the nucleotide position 811,370 on the genomic reference sequence NC_029544.1. We referred to this SNP as g.811370, and determined its genotypes in all 196 F_2_ offspring used in this study by PCR-RFLP analysis (Fig. S8). The result showed a full concordance between genotypes and plumage colour phenotypes (Fig. 2C and Table S4), and the *P*-value of Fisher’s exact test for case-control association was 1.1 × 10^−33^. Linkage analysis using LM2 confirmed that g.811370 forms a single linkage group with the remaining 5 markers in LGE22. g.811370 was located within a 32.6-cM interval between two flanking markers (Fig. 2B). The recombination rate between these flanking markers was 40.2 cM/Mb, which was much higher than the average recombination rate of 2.7 cM/Mb (Table S1). The extremely high rate of recombination in the causative region was likely attributable to sequence gaps in the reference genome assembly of quail LGE22. Sequence gaps were also predicted to exist in chicken LGE22 that also showed a high rate of recombination^34^.

To test whether the nonsense mutation was present in other quail strains besides the L strain, we performed PCR-RFLP analysis using a total of 40 quail of 10 strains that included L and WE strains (Fig. S9). The nine strains other than the L strain exhibited four types of plumage colour phenotypes that included wild type, black, whitish, and panda. The result of the PCR-RFLP analysis showed that the nonsense mutation was present only in the L strain (Fig. S9).

### Low expression level of the *PMEL* gene

**Figure 4:**
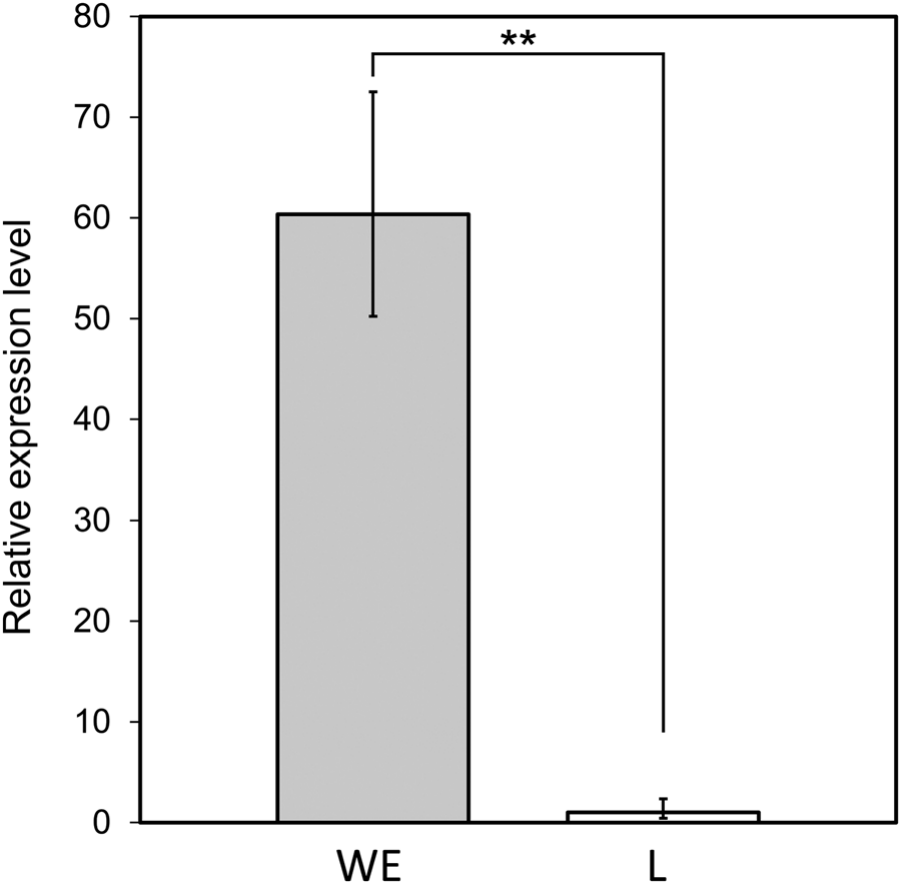
Comparison of the expression level of the *PMEL* gene between the WE and L strains. Bars indicate the average 2-ΔΔCT values with the standard deviation across three embryos. *PMEL* expression was much lower in mutants than in wild types (^**^P < 0.01).

In general, a premature stop codon causes mRNA degradation via a nonsense-mediated decay mechanism^35^. Therefore, we investigated the level of *PMEL* gene expression in the L strain. Expression levels of *PMEL* in 11-day-old whole embryos were compared between the L and WE strains: the expression level was significantly lower (<1/60) in the L strain than in the WE strain (Welch’s two-sided t-test, P < 0.05) (Fig. 4).

## Discussion

We have shown that a nonsense mutation in *PME*L is associated with the yellowish plumage of the L strain of the Japanese quail (Figs 2, 3, and S8). None of the ten quail strains except for the L strain had the nonsense mutation (Fig. S9). PMEL, a pigment cell-specific type I transmembrane glycoprotein, is a main component of the fibrillar matrix in melanosomes and is necessary for fibril formation in premelanosomes and for melanosome morphogenesis^31–33^. This protein is also referred to as PMEL17, SILVER, SILV, gp100, or ME20. PMEL consists of a large luminal domain, a carboxyl-terminal transmembrane, and cytoplasmic domains^31–33^. Post-translational modification and proteolytic processing of PMEL produce Mα and Mβ fragments^36–38^. The Mα fragment undergoes further proteolytic cleavage, and the resultant fragments oligomerise into fibrillar structures that assemble laterally into sheets^32^. The sheet-like matrix of PMEL fibrils is considered to serve as the scaffold for melanin polymerisation and deposition and also to function for sequestering reactive eumelanin intermediates that damage melanosomal contents, melanosome membranes, and cytosolic contents in melanocytes. PMEL fibrils also may facilitate intracellular and intercellular transport of eumelanin by producing aggregated particles of eumelanin polymers^31,32^. *Pmel*-knockout mice exhibit modest hypopigmentation, with reduced eumelanin content in hair and morphologically abnormal melanosomes; however, PMEL deficiency did not affect the production of pheomelanin and survival of pigment cells^39^. It should be noted that pheomelanosomes, which predominantly generate pheomelanin, contain neither PMEL fibrils nor intraluminal fibrils^31,32^. The expression level of *PMEL* in the L strain is only about 2% of that in the wild type and the truncated form of PMEL lacks the major part of the full-length protein. Furthermore, the plumage phenotype is consistent with what is expected for a null mutant of *PMEL*^39,40^. Thus, the nonsense mutation in *PMEL* in the L strain causes a loss of function. As observed for hair and skin hypopigmentation in the *Pmel*-knockout mice, the loss of *PMEL* in the L strain may suppress deposition of eumelanin, but not of pheomelanin, in feathers, which may lead to a diluted feather colour and result in yellowish plumage (Fig. 1). Thus, we propose that *PMEL* is the causative gene for the yellowish plumage. The loss of *PMEL* may affect processes of eumelanin deposition, such as production of eumelanin and transfer of eumelanin to keratinocytes.

*Pmel* deficiency has no apparent effect on the viability of pigment cells in mice^39^. The low expression of *PMEL* in the L strain is likely due to the nonsense-mediated mRNA degradation, leading to deactivation of *PMEL* function before translation. However, it should be noted that the low expression of mutant-type *PMEL* may be due to the reduced number of pigment cells owing to the impact of PMEL loss on cell viability or proliferation.

Hypopigmentation due to spontaneous mutations of *PMEL* has been reported in various vertebrate species, including zebrafish, chickens, dogs, horses, cattle, mice, and humans^9,41–48^. It is of interest to note that hypopigmentation is more severe in these mutants than those in the *Pmel*-knockout mouse and the L strain. Partial alternation of *PMEL* is associated with these severe hypopigmentation phenotypes, e.g., a missense mutation in horses (*Silver*), a retrotransposon insertion in dogs (*Merle*), an in-frame insertion in chickens (*Dominant white*), and a nonsense mutation in zebrafish (*fading vision*)^9,41–45^. These partial alterations may cause the production of pathogenic forms of PMEL, which may reduce the viability of melanocytes or integrity of melanocytes and result in severe hypopigmentation phenotypes^9,41–45^. We should note that modest hypopigmentation phenotypes in *PMEL*-null mutants might be attributable to a partial compensation by molecular mechanisms that have similar functions to PMEL fibrils.

To clarify how the yellowish plumage is caused by the nonsense mutation in *PMEL*, future studies should quantify melanin and analyse the morphology of melanosomes in the L strain. Developmental abnormalities in the eyes and optic nervous system should also be investigated because ocular abnormalities have been reported in *PMEL* mutants of dogs, horses, and fish^41–47^. In addition, for definitive identification of *PMEL* as the causative gene of the yellowish plumage, complementary tests should be conducted. The *Smoky* allele of *PMEL* is associated with a Smoky plumage in chickens^9^. This mutation gives rise to a greyish plumage phenotype in homozygotes by functioning as a null allele^40^. Generation of interspecific F_1_ hybrids between the L strain and chicken homozygote for the *Smoky* allele is a candidate method for complementation tests^21^.

In this study, we demonstrated that a novel yellowish plumage phenotype in the L strain of quail can be caused by the PMEL deficiency. Three different types of *PMEL* mutations have been reported in chickens^9^; however, *PMEL* mutation has not been reported in quail. Our present findings contribute to improved understanding of the genetic basis of plumage colour traits in birds. The L strain would be useful as a null mutant to help better understand the molecular basis of melanin deposition. Quail is a useful experimental animal owing to its features, such as small body size, short generation time, and disease and environmental resistance; however, molecular genetic analysis has been difficult in this species because of the absence of a draft genome sequence and difficulties in obtaining a sufficient number of SNPs that are required for an association test. Our present study suggests that ddRAD-seq is effective for identifying genes responsible for various hereditary traits in Japanese quail.

## Methods

### Animals, experimental cross, and phenotyping

A female of the L strain and a male of the WE strain were used as the parent generation to obtain the F_1_ generation offspring. Both quail strains are maintained as long-term closed colonies at the Avian Bioscience Research Center, Nagoya University. A total of 378 offspring in the F_2_ generation, which included embryos just before hatching, juveniles, and adults, were obtained by intercrossing between one F_1_ female and three F_1_ females. Of these F_2_ offspring, 96 males and 100 females were used for library construction for ddRAD-seq, and 181 were used for a case-control association test (Table S5). Plumage colour phenotypes of the F_2_ offspring were distinguishable by their appearance even in juveniles just after hatching. The gender of quail at sexual maturity (approximately 5 weeks) was judged by the colour pattern of feathers around the neck and chest: females show black spots on a pale brown background and males have reddish brown feathers. Quail were maintained with free access to water and a commercially available diet. The photoperiod was set at 14:10 h L:D, and room temperature was controlled at approximately 25 °C. Animal care and all experimental procedures were approved by the Animal Experiment Committee, Graduate School of Bioagricultural Sciences, Nagoya University (approval numbers: 2015030220), and the experiments were conducted according to Regulations on Animal Experiments at Nagoya University.

### Purification of genomic DNAs from blood samples

Blood samples were collected from isoflurane-anesthetized quails of the P, F_1_, and F_2_ generations and heparinized immediately. To wash blood cells, blood samples were mixed with an equal volume of 0.9% NaCl solution, and spun down at 1000 rpm for 1 minute at room temperature, and then supernatant was discarded. After washing three times, blood cells were stored at −20 °C until use. Genomic DNA was extracted from 2.5 μl of blood cells using the DNeasy Blood & Tissue kit (Qiagen, Valencia, CA, USA). DNA was quantified using the Qubit dsDNA BR Assay Kit (Thermo Fisher Scientific, Waltham, MA). Spectrophotometric assessment of nucleic acid purity was performed using a BioSpec-nanospectrophotometer (Shimadzu Corporation, Tokyo, Japan), and genomic DNA with an OD260/280 ratio of 1.8–2.0 and an OD260/230 ratio of around 2.0 was used for library construction. Genomic DNA was diluted to a final concentration of 20 ng/μl with 10 mM Tris-Cl, pH 8.5 and stored at −20 °C until use.

### Library construction

Genomic DNAs of the P and F_2_ generations were used for library construction according to a method described elsewhere^25^. After double-digestion of 100 ng DNA from each sample using EcoRI and MseI (New England BioLabs, Beverly, MA) in a single reaction, a P1 adaptor containing a 5-bp barcode sequence and a P2 adapter (Tables S6) were ligated to the ends of DNA fragments using T4 DNA Ligase (TaKaRa Bio Inc., Otsu, Shiga, Japan). After ligation, DNA samples were pooled at equimolar concentrations. DNA fragments ranging from 300 to 500 bp were collected by a Pippin Prep (Sage Science, USA), and purified using AMPure XP beads (Beckman Coulter, Inc., USA), and then amplified (six cycles) with PCR primer sets containing 6-bp index sequences, using a Phusion High-Fidelity DNA polymerase (New England Biolabs) (Table S7). Library quality was validated by a 2100 Bioanalyzer with Agilent high-sensitivity DNA kits (Agilent Technologies, Santa Clara, CA). The library was quantified by quantitative PCR using an Applied Biosystems 7500 Real Time PCR System with a KAPA library quantification kit (Kapa Biosystems, Woburn, MA USA). Each library was adjusted to 2 nM, and then the same amounts from each library were pooled to obtain the final library. In this study, three final libraries were constructed, and each was sequenced in one lane using HiSeq. 1500 (Illumina Inc., San Diego, CA) at the Functional Genomics Facility, National Institute for Basic Biology (NIBB). In the first sequencing using a library with 17 samples (two samples of parents, six samples of F_2_ offspring, and nine samples unrelated to this work), the library was sequenced with 101-bp paired-end readings, which yielded 155.5 million paired-end reads (31 Gb). In the second sequencing of the library with 96 samples (parents, 93 F_2_ males and a single F_2_ female), the library was sequenced with 106-bp paired-end readings, which yielded 199 million paired-end reads (42 Gb). In the third sequencing of the library with 96 samples of F_2_ females, the library was sequenced with 106-bp paired-end readings, which yielded 212.2 million paired-end reads (45 Gb). Paired-end reads that were aligned concordantly at one time accounted for 52–76% of the total paired-end reads.

### Detection of SNPs and generation of a genotype dataset for a case-control association test

SNPs were detected in the Illumina raw reads using the software pipeline Stacks version 1.44^26^. The data analysis procedures to detect SNPs and those to generate a genotype dataset for a case-control association test are described in Supplementary Note.

### Case-control association test and genetic mapping

A case-control association test was performed using Fisher’s exact test with PLINK ver. 1.90^49^. The R package ‘qqman’ was used to create a Manhattan plot^50^. The *yw* locus was placed within an interval between two markers, as the total distance of LGE22 became minimised.

### PCR-RFLP analysis

The site of the nonsense mutation can be recognized by StyI in the L strain, but not in the WE strain (Fig. S8). By using this difference, we performed PCR-RFLP analysis to determine genotypes at the site of mutation in F_2_ offspring. Besides F_2_ offspring, 10 quail strains, including L, WE, AMRP, Quv, RWN, rb-TKP, NIES-FR/French, W (Wild), JW, and Estonian, were analyzed^51–53^. We examined four individuals of each strain using genomic DNA that was extracted from whole blood. PCR was performed using a 25-μl reaction volume containing quail genomic DNA, 1 × AmpliTaq Gold® 360 PCR Master Mix (Thermo Fisher Scientific), and 2 μM of each primer. Primer sequences and PCR conditions are described in Table S8. After digestion in a 20 μl reaction mixture containing 5 μl of PCR product, 10 units of StyI-HF (New England Biolabs), and 1 × CutSmart Buffer for 6 h at 37 °C, PCR products were electrophoresed in 2% agarose gel, followed by visualization with ethidium bromide.

### Complimentary DNA synthesis, Sanger sequencing, and quantitative PCR analysis

According to a previous study^9^, in which 14-day-old whole chicken embryos were used for *PMEL* expression analysis, total RNA was isolated from 11-day-old whole embryos (three individuals of each strain), whose developmental stage were almost equivalent to 14-day-old chicken embryos^54^, using TRIZOL reagent (Thermo Fisher Scientific, Waltham, MA, USA). The gender of these embryos was undetermined. After treating total RNAs with RQ1 RNase-Free DNase (Promega Corporation, Madison, WI, USA,), complimentary DNA (cDNA) was synthesised using Super ScriptIII Reverse Transcriptase (Thermo Fisher Scientific) with Oligo dT primers. Coding sequences of *PMEL* were amplified using KOD FX Neo (TOYOBO, Osaka, Japan). PCR products were electrophoresed in agarose gel and purified using a QIAquick Gel Extraction Kit (Qiagen, Valencia, CA, USA). Purified DNA fragments were sequenced by an ABI PRISM 3130 Analyzer (Thermo Scientific). Relative expression levels were assayed using a Fast SYBR Green Master Mix (Thermo Scientific) on a StepOnePlus Real-Time PCR System (Thermo Scientific) in three samples of each strain. Amplification efficiencies of primer pairs specific for the normalising control *ACTB* and *PMEL* were 80 and 116%, respectively. All reactions were conducted in triplicate. For all genes, single peaks were found in the melting curves. Cycle thresholds (CT) were generated with the StepOne software (Thermo Scientific) and analysed using the comparative CT method^55^. Primer sequences and PCR reaction conditions are reported in Table S8.

### Statistical analysis

Whether the yellowish plumage is inherited in an autosomal recessive manner was tested by Pearson’s chi-squared goodness of fit tests (α = 0.05). Comparison of gene expression (ΔCTs) was performed using two-tailed Welch’s t-test, with a two-sided alternative hypothesis (α = 0.01). Segregation distortion was tested by Pearson’s chi-squared goodness of fit tests with a Bonferroni correction (α = 0.001). Sample sizes for each analysis were chosen based on prior experience.

## Electronic supplementary material

Supplementary Information

## Data Availability

The Illumina data generated in this study have been deposited in the DDBJ Sequence Read Archive (DRA) (accession codes DRA005969). Sequence data of quail *PMEL* were deposited in DDBJ (accession codes LC309103 and LC309104). The final and intermediary genotype dataset for construction of the genetic map and for other miscellaneous information are available from the corresponding author upon request.
