# Supplementary material for: Nonsense mutation in PMEL is associated with yellowish plumage colour phenotype in Japanese quail
Source: Sci Rep. 2018 Nov 13;8:16732. doi: 10.1038/s41598-018-34827-4 (PMC6233202; doi:10.1038/s41598-018-34827-4)
Supplement: Supplementary file 1 — Supplementary Information [file 41598_2018_34827_MOESM1_ESM.pdf]

Supplementary Information

Nonsense mutation in PMEL is associated with yellowish plumage colour phenotype in Japanese quail

Satoshi Ishishita[1†], Mayuko Takahashi[2†], Katsushi Yamaguchi[3], Keiji Kinoshita[1], Mikiharu Nakano[1], Mitsuo Nunome[1], Shumpei Kitahara[2], Shoji Tatsumoto[4], Yasuhiro Go[4,5], Shuji Shigenobu[3], Yoichi Matsuda[1,2*]

[1]Avian Bioscience Research Center, Graduate School of Bioagricultural Sciences, Nagoya University, Furo-cho, Chikusa-ku, Nagoya, Aichi, 464-8601, Japan

[2]Laboratory of Animal Genetics, Department of Applied Molecular Biosciences, Graduate School of Bioagricultural Sciences, Nagoya University, Furo-cho, Chikusa-ku, Nagoya, Aichi, 464-8601, Japan

[3]Functional Genomics Facility, National Institute for Basic Biology, Okazaki, Aichi 444-8585, Japan

[4]Exploratory Research Center on Life and Living Systems (ExCELLS), National Institutes of Natural Sciences, Okazaki, Aichi 444-8585, Japan

[5]National Institute for Physiological Sciences, Okazaki, Aichi 444-8585, Japan

[†] These authors contributed equally to this work

* Corresponding author.

Supplementary note

Analysis of paired-end reads by Stacks

The sequence data were demultiplexed by barcode, cleaned by removing reads of low quality, and shortened to 95 bp (1st run) or 100 bp (2nd and 3rd runs) using the Stacks program 'process_radtags' (-c -q –r --adapter_mm 1 -t 100). The processed reads were aligned to the *Coturnix japonica* 2.0 (GCF_001577835.1) draft genome using the program Bowtie 2 ver. 2.2.8 (Langmead *et al.*, 2009) with default parameters. Only paired reads aligned concordantly to the reference genome at one time were analysed by the Stacks program 'ref_map.pl'.

The 'ref_map.pl' executes several programs: (i) 'pstack', which builds loci according to the alignment position of each read; (ii) 'cstacks', which creates catalogues of all loci for the parents and F_2 offspring; and (iii) 'sstcks', which matches each catalogue of loci of F_2 offspring against catalogues of the parents. The parameters of ref_map.pl were set as the default except for the number of mismatches allowed between sample loci when build the catalog (-n = 3). The minimum depth of coverage to report a stack in pstacks was three (-m = 3). The Stacks program 'genotypes' was executed to export the genotype dataset for the F_2 offspring. The parameters of 'genotype' were set as the default: minimum number of progeny required to print a marker (-r = 1). This outputted a genotype dataset of parents and 196 F_2 offspring, which contained genotypes at a total of 100,613 markers, although genotype data were missing at many sites in the data set.

Procedure for selection of Z-chromosome-linked markers

To select Z chromosome-linked markers, the genotype data were divided by gender after premapping quality control procedures (i) and (ii). In F_1 progeny, the two Z chromosomes in males are derived from both parental strains, and either of these chromosomes is transmitted to F_2 males and females; however, a single Z chromosome in females is derived from the paternal WE strain male and is only transmitted to F_2 males, except for the pseudoautosomal region (PAR) (markers

on the PAR were not identified in this study). Therefore, we searched markers showing heterozygosity or homozygosity for the WE strain-type allele in >90% of F_2 males, and those showing either the L or WE strain-type allele in >90% F_2 females.

Generation of a genotype dataset for a case-control association test

SNP markers and individuals used for a case-control association test were selected in the following manner. To eliminate markers located on highly repetitive DNA sequences, those showing more than 1,500 of coverage depth per individual were eliminated. Loci with a minimum coverage (eight in this study) in each individual, which consisted of one or two alleles with a given minimum coverage (three in this study) in each allele, were used for genotyping. This filtering was performed using a custom R script, based on marker depth output by 'sstacks' of the Stacks program. Markers that were not assigned to known chromosomes or linkage groups were eliminated from the genotype data set. The dataset containing genotypes at 10,498 markers were yielded by the filtering.

The genotype data set underwent premapping quality control (van Ooijen and Jansen, 2013), which excluded (i) individuals with a lot of missing data (>70% in this study); (ii) markers with a lot of missing data (>5% in this study); and (iii) markers with highly significant segregation distortion (SD) ($P < 0.001$ with Bonferroni correction in this study). After excluding markers with highly significant SD, we selected candidate Z-linked markers that were shared between males and females. After these filtering steps, the coverage depth of each genotype in the F_2 offspring was more than 7 and less than 250.

The genotype data set were analysed by the software Lep-MAP2 (LM2), which can construct genetic maps from a large data set (Rastas *et al.*, 2015). Three modules of LM2, SeparateChromosomes, Joinsingles, and OrderMarkers, were used to construct a genetic map. The SeparateChromosomes module assigns markers into LGs by calculating all pair-wise LOD scores

between markers and subsequently joins markers with LOD scores higher than a given parameter 'lodLimit' (LodLimit=20 in this study). The JoinSingles module assigns singular markers into existing LGs by calculating LOD scores between each singlular marker and markers in the existing LGs and joins markers with LOD scores higher than a given parameter (LodLimit=10 in this study). The OrderMarkers module orders markers within each LG as the likelihood of the data becomes maximized. We arranged autosomal markers into 33 LGs using the SeparateChromosomes module. The minimum size of LGs was set as 2 (sizeLimit=2). Two autosomal markers were not assigned into any LGs by the SeparateChromosome module, and these markers were also not assigned into any LGs by the JoinSingles module. Markers within the 33 LGs were ordered using the OrderMarkers module. This module was used with options to set the number of iteration of calculation (numMergeIterations=10) and to calculate sex averaged genetic distances between adjacent markers with the Kosambi mapping function (sexAveraged=1, useKosambi=1). Z-linked markers were analysed by LM2 independently of autosomal markers by the same procedure as that for autosomal markers except for two options in the OrderMarkers module (initRecombination=0.05 0.000000001 learnRecombinationParameters=1 0), which were required for computation of the marker order and distances between markers without recombination in female meiosis. A genetic map consisting of a total of 34 linkage groups was constructed at this step.

Markers with similar or identical genotypes, which were located on an identical position on the genetic map, were eliminated from the genotype dataset. Markers mapped to chromosome 1 were grouped into two different LGs. A similar separation of linkage groups on the same chromosome occurred for markers mapped to chromosome 2 and LGE22. These separated marker groups were joined manually into single groups. To correct the genetic map, we searched for markers showing >5-cM genetic distance between adjacent markers, and then apparently erroneous marker ordering was corrected by rearranging markers or excluding markers with a high frequency of double recombination. After eliminating markers with highly significant SD,

the final genetic map was constructed. Distances between markers in the final genotype dataset were recalculated by Map Manager QTX ver. 0.30 (Manly *et al.*, 2001) with the Kosambi mapping function. The genetic map was visualised by a custom R script.

References

Langmead, B., Trapnell, C., Pop, M. & Salzberg, S. L. Ultrafast and memory-efficient alignment of short DNA sequences to the human genome. *Genome Biol.* **10,** R25 (2009).

van Ooijen JW & Jansen J (2013) *Genetic mapping in experimental populations.* (Cambridge University Press, Cambridge, U.K., 2013).

Rastas, P., Calboli, F. C. F., Guo, B., Shikano, T. & Merilä, J. Construction of ultradense linkage maps with Lep-MAP2: stickleback F2 recombinant crosses as an example. *Genome Biol. Evol.* **8,** 78–93 (2015).

Manly, K. F., Cudmore, R. H. & Meer, J. M. Map Manager QTX, cross-platform software for genetic mapping. *Mamm. Genome.* **12,** 930–932 (2001).

Table S1. Summary of genetic map.

Chr.	Size of chr.[a] (Mb)	No. of markers	Length (cM)	Average of intermarker distance (cM)	Max. of intermarker distance (cM)	Proximal[b] (Mb)	Distal[c] (Mb)	Proximal–Distal[d] (Mb)	Covered region[e] (%)	cM/Mb [f]	Average of intermarker distance (Mb)
1	175.66	331	316.9	1	30.8	0.25	174.76	174.51	99.3	1.8	0.5
2	134.82	266	253	1	31.6	0.87	134.64	133.76	99.2	1.9	0.5
3	100.94	220	181	0.8	5.8	0.13	100.22	100.09	99.2	1.8	0.5
4	82.19	138	131.9	1	6.5	1.59	81.98	80.39	97.8	1.6	0.6
5	54.02	124	114.8	0.9	4.9	1.22	53.93	52.71	97.6	2.2	0.4
6	31.42	49	62.9	1.3	7	2.04	30.66	28.62	91.1	2.2	0.6
7	33.42	60	79.4	1.3	5.7	0.18	33.27	33.09	99	2.4	0.6
8	26.89	87	61	0.7	2.9	0.11	25.57	25.46	94.7	2.4	0.3
9	21.33	78	63.2	0.8	3.5	0.7	19.83	19.13	89.7	3.3	0.2
10	18.48	66	60.1	0.9	5.3	0.63	17.54	16.91	91.5	3.6	0.3
11	17.8	57	51.7	0.9	5.3	3.32	17.68	14.36	80.7	3.6	0.3
12	17.22	56	62.3	1.1	4.8	0.13	16.89	16.76	97.3	3.7	0.3
13	15.71	52	56.7	1.1	4.6	0.58	15.39	14.81	94.3	3.8	0.3
14	12.83	51	60.6	1.2	6.2	1.55	12.53	10.98	85.6	5.5	0.2
15	11.7	41	48.6	1.2	4.1	1.53	10.6	9.07	77.5	5.4	0.2
16	0.35	2	0.3	0.3	0.3	0.25	0.28	0.03	8.6	10	-
17	8.97	21	53	2.6	14.1	0.35	8.33	7.98	89	6.6	0.4
18	9.55	38	55.7	1.5	12.4	0.1	8.44	8.34	87.3	6.7	0.2
19	8.76	16	36.2	2.4	8.7	0.99	5.04	4.05	46.2	8.9	0.3
20	12.45	42	53.1	1.3	6	0.45	12.35	11.9	95.6	4.5	0.3
21	5.95	25	69.5	2.9	12	0.17	5.56	5.39	90.6	12.9	0.2
22	4.13	15	59	4.2	11.4	0.19	3.96	3.76	91	15.7	0.3
23	5.05	17	48.5	3	13.4	0.71	4.03	3.32	65.7	14.6	0.2
24	5.5	22	52.2	2.5	7.4	0.35	5.47	5.12	93.1	10.2	0.2
25	2.51	13	26	2.2	7.6	0.81	1.89	1.08	43	24.1	0.1
26	4.93	20	54.8	2.9	21.3	0.79	4.49	3.7	75.1	14.8	0.2
27	4.78	13	43	3.6	13	1.66	4.1	2.43	50.8	17.7	0.2
28	4.02	22	55.1	2.6	13.9	0.33	3.95	3.62	90	15.2	0.2
LGE22	1.78	5	55.1	13.8	44.6	0.26	1.31	1.04	58.4	53	0.2
LGE64	0.58	2	0.3	0.3	0.3	0.34	0.37	0.03	5.2	10	-
Z	67	55	87.7	1.6	6.4	0.3	66.44	66.14	98.7	1.3	1.2
Overall	900.74	2004	2353.6	1.2	44.6	-	-	858.58	95.3	2.7	0.4

[a] The size of each chromosome or LG in *Coturnix japonica* 2.0.

[b] The nearest marker proximal to the start site.

[c] The farthest marker distal to the start site.

[d] The distance between the most proximal and distal markers.

[e] Percentage of distance between the most proximal and distal makers in the total length of each chromosome or LG.

[f] Recombination rate.

Table S2. Recombination fractions and LOD scores between the *yw* locus and all markers in LGE22.

SNPs	Recombination fractions	LOD scores
295443	0.122	11.48
295438	0.119	11.97
295380	0.093	14.33
g.811370	0.000	26.49
295244	0.159	9.75
295306	0.227	6.01

Recombination fractions and LOD scores were estimated using genotype data of homozygous mutant individuals of F_2 hybrids.

Table S3. Genes within the causative genomic region.

Gene ID	Symbol	Description	Start_position	End_position	Orientation
107325708	DIP2B	disco interacting protein 2 homolog B	206942	266333	minus
107325712	LARP4	La ribonucleoprotein domain family member 4	266937	284550	minus
107325701	LIMA1	LIM domain and actin binding 1	292071	315762	plus
107325707	LOC107325707	uncharacterized LOC107325707	292278	317246	minus
107325704	CERS5	ceramide synthase 5	317280	335184	plus
107325706	LOC107325706	cytochrome c oxidase assembly protein COX14	336421	338089	minus
107325705	GPD1	glycerol-3-phosphate dehydrogenase 1	338201	342954	minus
107325703	SMARCD1	SWI/SNF related, matrix associated, actin dependent regulator of chromatin, subfamily d, member 1	343642	350812	minus
107325723	ASIC1	acid sensing ion channel subunit 1	355661	371556	minus
107325722	RACGAP1	Rac GTPase activating protein 1	384141	391976	plus
107325714	HDAC7	histone deacetylase 7	403795	460106	plus
107325715	LOC107325715	twist-related protein 2-like	460865	463774	minus
107325720	SLC48A1	solute carrier family 48 member 1	474347	484037	minus
107325717	RAPGEF3	Rap guanine nucleotide exchange factor 3	494853	509573	plus
107325719	ENDOU	endonuclease, poly(U) specific	511293	517955	plus
107325700	NEUROD4	neuronal differentiation 4	527600	532542	minus
107325698	LOC107325698	protein SUPPRESSOR OF GENE SILENCING 3 homolog	548879	550115	minus
107325693	TESPA1	thymocyte expressed, positive selection associated 1	550109	561401	minus
107325732	PPP1R1A	protein phosphatase 1 regulatory inhibitor subunit 1A	571249	579751	plus
107325730	PDE1B	phosphodiesterase 1B	582550	593872	minus
107325731	NCKAP1L	NCK associated protein 1 like	607006	620937	minus
107325729	GTSF1	gametocyte specific factor 1	621819	630935	plus
107325740	ARF3	ADP ribosylation factor 3	682361	683701	minus
107325739	WNT1	Wnt family member 1	688047	696008	plus
107325757	PRKAG1	protein kinase AMP-activated non-catalytic subunit gamma 1	707820	714298	minus
107325738	KMT2D	lysine methyltransferase 2D	718999	749252	minus
107325761	DHH	desert hedgehog	751775	756381	minus
107325760	LMBR1L	limb development membrane protein 1 like	757266	762451	minus
107325742	LOC107325742	tubulin alpha-1A chain	764872	785403	minus
107325741	LOC107325741	tubulin alpha-1B chain-like	764872	777003	minus
107325734	LOC107325734	tubulin alpha-8 chain pseudogene	770887	772927	minus
107325743	LOC107325743	tubulin alpha-1C chain	789186	793596	plus
107325758	LOC107325758	transmembrane protein 198-like	794116	797862	plus
107325759	PYM1	PYM homolog 1, exon junction complex associated factor	797693	800937	minus
107325737	DGKA	diacylglycerol kinase alpha	801183	807808	plus
107325756	PMEL	premelanosome protein	807870	812723	minus
107325763	CDK2	cyclin dependent kinase 2	814942	817700	plus
107325751	RAB5B	RAB5B, member RAS oncogene family	818929	825469	plus
107325750	SUOX	sulfite oxidase	825526	827907	plus
107325762	IKZF4	IKAROS family zinc finger 4	829130	839186	plus
107325764	RPS26	ribosomal protein S26	841577	843447	plus
107325755	ERBB3	erb-b2 receptor tyrosine kinase 3	846972	858448	plus
107325747	PA2G4	proliferation-associated 2G4	859367	868729	plus
107325749	LOC107325749	uncharacterized LOC107325749	859963	868872	minus
107325748	ZC3H10	zinc finger CCCH-type containing 10	871419	873594	plus
107325736	ESYT1	extended synaptotagmin 1	876755	879154	plus
107325752	SMARCC2	SWI/SNF related, matrix associated, actin dependent regulator of chromatin subfamily c member 2	889385	898653	minus
107325754	RNF41	ring finger protein 41	899656	907901	minus
107325735	NABP2	nucleic acid binding protein 2	907985	909946	plus
107325733	SLC39A5	solute carrier family 39 member 5	911556	918885	plus
107325745	ANKRD52	ankyrin repeat domain 52	922711	941702	minus
107325746	LOC107325746	coenzyme Q-binding protein COQ10 homolog A, mitochondrial-like	942403	944238	plus
107325809	R3HDM2	R3H domain containing 2	944609	965864	minus
107325784	LOC107325784	inhibin beta C chain-like	971817	973972	plus
107325767	LOC107325767	inhibin beta E chain-like	980353	983544	plus
107325822	GLI1	GLI family zinc finger 1	985756	998034	plus
107325810	DNAJC14	DnaJ heat shock protein family (Hsp40) member C14	998591	1001299	plus
107325811	ORMDL2	ORMDL sphingolipid biosynthesis regulator 2	1001476	1003752	minus
107325801	SARNP	SAP domain containing ribonucleoprotein	1003892	1024329	plus
107325827	GDF11	growth differentiation factor 11	1030586	1036964	minus
107325828	INPP1	inositol polyphosphate-1-phosphatase	1037987	1039935	plus
107325768	CD63	CD63 molecule	1039806	1041161	plus
107325819	RDH5	retinol dehydrogenase 5	1041381	1044165	minus
107325769	BLOC1S1	biogenesis of lysosomal organelles complex 1 subunit 1	1044226	1045385	minus
107325796	ITGA7	integrin subunit alpha 7	1047040	1059350	plus
107325799	MCRS1	microspherule protein 1	1059366	1063159	plus
107325798	SPATS2	spermatogenesis associated serine rich 2	1063291	1082406	minus

Table S4. Plumage colour phenotypes and genotypes at the site of the candidate mutation in F_2 offspring.

Individual ID	Phenotype	Genotype	Individual ID	Phenotype	Genotype	Individual ID	Phenotype	Genotype
419	Yellowish	L	3812	Wild type	H	3001	Wild type	W
421	Yellowish	L	3813	Yellowish	L	3002	Wild type	W
423	Wild type	W	3816	Wild type	W	3006	Wild type	H
425	Wild type	H	3817	Wild type	W	3007	Wild type	H
427	Wild type	H	3822	Wild type	H	3008	Wild type	W
430	Wild type	W	3824	Wild type	H	3013	Wild type	W
2982	Wild type	H	4010	Wild type	H	3195	Wild type	H
2984	Wild type	W	4011	Wild type	H	3196	Wild type	H
2986	Yellowish	L	4014	Wild type	W	3198	Yellowish	L
2988	Wild type	H	4015	Wild type	W	3200	Yellowish	L
2989	Wild type	W	4017	Wild type	H	3201	Yellowish	L
2990	Yellowish	L	4019	Yellowish	L	3209	Yellowish	L
2991	Wild type	H	4023	Yellowish	L	3211	Wild type	H
2996	Yellowish	L	4024	Wild type	H	3385	Wild type	W
3003	Wild type	H	4025	Wild type	H	3389	Yellowish	L
3004	Wild type	W	4031	Wild type	H	3392	Yellowish	L
3005	Wild type	W	4032	Wild type	W	3394	Wild type	W
3009	Wild type	W	4033	Wild type	W	3395	Wild type	H
3010	Yellowish	L	4037	Wild type	H	3396	Wild type	W
3012	Yellowish	L	4038	Wild type	W	3397	Wild type	H
3193	Wild type	W	4168	Yellowish	L	3398	Wild type	H
3194	Wild type	H	4169	Wild type	H	3400	Wild type	H
3197	Wild type	W	4170	Wild type	H	3402	Wild type	H
3199	Wild type	H	4171	Wild type	W	3403	Wild type	H
3203	Yellowish	L	4173	Yellowish	L	3404	Yellowish	L
3204	Wild type	H	4174	Wild type	H	3407	Yellowish	L
3206	Yellowish	L	4175	Wild type	H	3408	Wild type	H
3207	Yellowish	L	4176	Wild type	H	3409	Yellowish	L
3210	Wild type	H	4179	Yellowish	L	3411	Wild type	H
3213	Yellowish	L	4180	Yellowish	L	3412	Wild type	H
3214	Wild type	H	4181	Wild type	W	3414	Wild type	H
3215	Yellowish	L	4182	Wild type	H	3415	Wild type	H
3216	Wild type	W	4185	Wild type	H	3530	Wild type	H
3217	Wild type	H	9999	Yellowish	L	3532	Wild type	W
3219	Wild type	H	499	Wild type	H	3535	Wild type	H
3221	Wild type	W	500	Yellowish	L	3538	Yellowish	L
3388	Wild type	H	530	Wild type	H	3542	Wild type	H
3390	Wild type	H	537	Wild type	H	3551	Wild type	W
3393	Yellowish	L	539	Yellowish	L	3800	Wild type	H
3401	Wild type	W	545	Wild type	H	3804	Yellowish	L
3405	Wild type	W	2733	Wild type	H	3805	Yellowish	L
3406	Wild type	H	2734	Wild type	H	3808	Wild type	H
3413	Wild type	H	2744	Wild type	H	3811	Wild type	H
3531	Wild type	H	2748	Wild type	H	3814	Yellowish	L
3533	Yellowish	L	2750	Wild type	H	3815	Wild type	W
3534	Wild type	W	2753	Wild type	H	3818	Wild type	H
3536	Wild type	H	2754	Wild type	W	3820	Wild type	H
3539	Wild type	H	2755	Wild type	H	3821	Wild type	H
3540	Wild type	H	2756	Yellowish	L	3823	Wild type	H
3541	Wild type	H	2757	Wild type	H	4012	Wild type	H
3543	Wild type	H	2758	Wild type	H	4013	Wild type	H
3544	Yellowish	L	2759	Wild type	H	4018	Wild type	W
3545	Yellowish	L	2761	Wild type	W	4022	Wild type	W
3546	Wild type	W	2762	Wild type	W	4027	Wild type	H
3547	Wild type	W	2763	Wild type	H	4028	Yellowish	L
3548	Wild type	W	2764	Yellowish	L	4030	Wild type	W
3549	Wild type	W	2981	Wild type	W	4034	Wild type	W
3550	Wild type	H	2987	Wild type	W	4036	Yellowish	L
3552	Wild type	H	2992	Wild type	H	4039	Wild type	W
3554	Yellowish	L	2993	Yellowish	L	4040	Wild type	W
3798	Wild type	H	2994	Wild type	H	4041	Wild type	H
3799	Wild type	H	2995	Yellowish	L	4172	Wild type	H
3801	Yellowish	L	2997	Wild type	H	4177	Wild type	H
3802	Wild type	W	2998	Wild type	W	4183	Wild type	H
3806	Wild type	H	2999	Wild type	H			
3810	Wild type	H	3000	Wild type	W			

L, L/L; W, +/+; H, Heterozygotes.

Table S5. Summary of samples.

Individual ID	Sequence sample ID	Generation	Gender	Plumage colour	Run number	Individual ID	Sequence sample ID	Generation	Gender	Plumage colour	Run number	Individual ID	Sequence sample ID	Generation	Gender	Plumage colour	Run number
8358	idx1_Y01_8358P	P	Female	Yellowish	1	8358	idx01_Y01_P8358	P	Female	Yellowish	2	499	idx01_Y01_01	F_2	Female	Wild type	3
7388	idx2_Y02_7388P	P	Male	Wild type	1	7388	idx01_Y02_P7388	P	Male	Wild type	2	500	idx01_Y02_02	F_2	Female	Yellowish	3
419	idx3_Y03_419M	F_2	Male	Yellowish	1	2982	idx01_Y03_3M	F_2	Male	Wild type	2	530	idx01_Y03_03	F_2	Female	Wild type	3
421	idx4_Y04_421M	F_2	Male	Yellowish	1	2984	idx01_Y04_4M	F_2	Male	Wild type	2	537	idx01_Y04_04	F_2	Female	Wild type	3
423	idx5_Y05_423M	F_2	Male	Wild type	1	2986	idx01_Y05_5M	F_2	Male	Yellowish	2	539	idx01_Y05_05	F_2	Female	Yellowish	3
425	idx6_Y06_425F	F_2	Female	Wild type	1	2988	idx01_Y06_6M	F_2	Male	Wild type	2	545	idx01_Y06_06	F_2	Female	Wild type	3
427	idx7_Y07_427F	F_2	Female	Wild type	1	2989	idx01_Y07_7M	F_2	Male	Wild type	2	2733	idx01_Y07_07	F_2	Female	Wild type	3
430	idx8_Y08_430F	F_2	Female	Wild type	1	2990*	idx01_Y08_8M	F_2	Male	Yellowish	2	2734	idx01_Y08_08	F_2	Female	Wild type	3
						2991	idx01_Y09_9M	F_2	Male	Wild type	2	2744	idx01_Y09_09	F_2	Female	Wild type	3
						2996	idx01_Y10_10M	F_2	Male	Yellowish	2	2748	idx01_Y10_10	F_2	Female	Wild type	3
						3003*	idx01_Y13_11M	F_2	Male	Wild type	2	2750*	idx01_Y13_11	F_2	Female	Wild type	3
						3004	idx01_Y17_12M	F_2	Male	Wild type	2	2753	idx01_Y17_12	F_2	Female	Wild type	3
						3005	idx01_Y18_13M	F_2	Male	Wild type	2	2754	idx01_Y18_13	F_2	Female	Wild type	3
						3009	idx01_Y19_14M	F_2	Male	Wild type	2	2755	idx01_Y19_14	F_2	Female	Wild type	3
						3010	idx01_Y28_15M	F_2	Male	Yellowish	2	2756	idx01_Y28_15	F_2	Female	Yellowish	3
						3012	idx01_Y29_16M	F_2	Female	Yellowish	2	2757	idx01_Y29_16	F_2	Female	Wild type	3
						3193	idx05_Y01_17M	F_2	Male	Wild type	2	2758	idx05_Y01_17	F_2	Female	Wild type	3
						3194	idx05_Y02_18M	F_2	Male	Wild type	2	2759	idx05_Y02_18	F_2	Female	Wild type	3
						3197	idx05_Y03_19M	F_2	Male	Wild type	2	2761*	idx05_Y03_19	F_2	Female	Wild type	3
						3199	idx05_Y04_20M	F_2	Male	Wild type	2	2762*	idx05_Y04_20	F_2	Female	Wild type	3
						3203	idx05_Y05_21M	F_2	Male	Yellowish	2	2763	idx05_Y05_21	F_2	Female	Wild type	3
						3204	idx05_Y06_22M	F_2	Male	Wild type	2	2764	idx05_Y06_22	F_2	Female	Yellowish	3
						3206	idx05_Y07_23M	F_2	Male	Yellowish	2	2981	idx05_Y07_23	F_2	Female	Wild type	3
						3207	idx05_Y08_24M	F_2	Male	Yellowish	2	2987	idx05_Y08_24	F_2	Female	Wild type	3
						3210	idx05_Y09_25M	F_2	Male	Wild type	2	2992	idx05_Y09_25	F_2	Female	Wild type	3
						3213	idx05_Y10_26M	F_2	Male	Yellowish	2	2993*	idx05_Y10_26	F_2	Female	Yellowish	3
						3214	idx05_Y13_27M	F_2	Male	Wild type	2	2994*	idx05_Y13_27	F_2	Female	Wild type	3
						3215	idx05_Y17_28M	F_2	Male	Yellowish	2	2995	idx05_Y17_28	F_2	Female	Yellowish	3
						3216	idx05_Y18_29M	F_2	Male	Wild type	2	2997	idx05_Y18_29	F_2	Female	Wild type	3
						3217	idx05_Y19_30M	F_2	Male	Wild type	2	2998	idx05_Y19_30	F_2	Female	Wild type	3
						3219	idx05_Y28_31M	F_2	Male	Wild type	2	2999	idx05_Y28_31	F_2	Female	Wild type	3
						3221*	idx05_Y29_32M	F_2	Male	Wild type	2	3000	idx05_Y29_32	F_2	Female	Wild type	3
						3388	idx06_Y01_33M	F_2	Male	Wild type	2	3001	idx06_Y01_33	F_2	Female	Wild type	3
						3390	idx06_Y02_34M	F_2	Male	Wild type	2	3002	idx06_Y02_34	F_2	Female	Wild type	3
						3393	idx06_Y03_35M	F_2	Male	Yellowish	2	3006*	idx06_Y03_35	F_2	Female	Wild type	3
						3401	idx06_Y04_36M	F_2	Male	Wild type	2	3007	idx06_Y04_36	F_2	Female	Wild type	3
						3405	idx06_Y05_37M	F_2	Male	Wild type	2	3008	idx06_Y05_37	F_2	Female	Wild type	3
						3406	idx06_Y06_38M	F_2	Male	Wild type	2	3013	idx06_Y06_38	F_2	Female	Wild type	3
						3413	idx06_Y07_39M	F_2	Male	Wild type	2	3195	idx06_Y07_39	F_2	Female	Wild type	3
						3531	idx06_Y08_40M	F_2	Male	Wild type	2	3196	idx06_Y08_40	F_2	Female	Wild type	3
						3533	idx06_Y09_41M	F_2	Male	Yellowish	2	3198	idx06_Y09_41	F_2	Female	Yellowish	3
						3534	idx06_Y10_42M	F_2	Male	Wild type	2	3200	idx06_Y10_42	F_2	Female	Yellowish	3
						3536	idx06_Y13_43M	F_2	Male	Wild type	2	3201	idx06_Y13_43	F_2	Female	Yellowish	3
						3539	idx06_Y17_44M	F_2	Male	Wild type	2	3209	idx06_Y17_44	F_2	Female	Yellowish	3
						3540	idx06_Y18_45M	F_2	Male	Wild type	2	3211	idx06_Y18_45	F_2	Female	Wild type	3
						3541	idx06_Y19_46M	F_2	Male	Wild type	2	3385	idx06_Y19_46	F_2	Female	Wild type	3
						3543	idx06_Y28_47M	F_2	Male	Wild type	2	3389	idx06_Y28_47	F_2	Female	Yellowish	3
						3544	idx06_Y29_48M	F_2	Male	Yellowish	2	3392	idx06_Y29_48	F_2	Female	Yellowish	3
						3545	idx08_Y01_49M	F_2	Male	Yellowish	2	3394	idx08_Y01_49	F_2	Female	Yellowish	3
						3546	idx08_Y02_50M	F_2	Male	Wild type	2	3395	idx08_Y02_50	F_2	Female	Wild type	3
						3547	idx08_Y03_51M	F_2	Male	Wild type	2	3396	idx08_Y03_51	F_2	Female	Wild type	3
						3548	idx08_Y04_52M	F_2	Male	Wild type	2	3397	idx08_Y04_52	F_2	Female	Wild type	3
						3549	idx08_Y05_53M	F_2	Male	Wild type	2	3398	idx08_Y05_53	F_2	Female	Wild type	3
						3550	idx08_Y06_54M	F_2	Male	Wild type	2	3400	idx08_Y06_54	F_2	Female	Wild type	3
						3552	idx08_Y07_55M	F_2	Male	Wild type	2	3402	idx08_Y07_55	F_2	Female	Wild type	3
						3554*	idx08_Y08_56M	F_2	Male	Yellowish	2	3403	idx08_Y08_56	F_2	Female	Wild type	3
						3798	idx08_Y09_57M	F_2	Male	Wild type	2	3404	idx08_Y09_57	F_2	Female	Yellowish	3
						3799	idx08_Y10_58M	F_2	Male	Wild type	2	3407	idx08_Y10_58	F_2	Female	Yellowish	3
						3801	idx08_Y13_59M	F_2	Male	Yellowish	2	3408	idx08_Y13_59	F_2	Female	Wild type	3
						3802	idx08_Y17_60M	F_2	Male	Wild type	2	3409	idx08_Y17_60	F_2	Female	Yellowish	3
						3806	idx08_Y18_61M	F_2	Male	Wild type	2	3411	idx08_Y18_61	F_2	Female	Wild type	3
						3810	idx08_Y19_62M	F_2	Male	Wild type	2	3412	idx08_Y19_62	F_2	Female	Wild type	3
						3812	idx08_Y28_63M	F_2	Male	Wild type	2	3414	idx08_Y28_63	F_2	Female	Wild type	3
						3813	idx08_Y29_64M	F_2	Male	Yellowish	2	3415	idx08_Y29_64	F_2	Female	Wild type	3
						3816	idx11_Y01_65M	F_2	Male	Wild type	2	3530	idx11_Y01_65	F_2	Female	Wild type	3
						3817	idx11_Y02_66M	F_2	Male	Wild type	2	3532	idx11_Y02_66	F_2	Female	Wild type	3
						3822	idx11_Y03_67M	F_2	Male	Wild type	2	3535	idx11_Y03_67	F_2	Female	Wild type	3
						3824	idx11_Y04_68M	F_2	Male	Wild type	2	3538	idx11_Y04_68	F_2	Female	Yellowish	3
						4010	idx11_Y05_69M	F_2	Male	Wild type	2	3542	idx11_Y05_69	F_2	Female	Wild type	3
						4011*	idx11_Y06_70M	F_2	Male	Wild type	2	3551	idx11_Y06_70	F_2	Female	Wild type	3
						4014	idx11_Y07_71M	F_2	Male	Wild type	2	3800	idx11_Y07_71	F_2	Female	Wild type	3
						4015	idx11_Y08_72M	F_2	Male	Wild type	2	3804	idx11_Y08_72	F_2	Female	Yellowish	3
						4017	idx11_Y09_73M	F_2	Male	Wild type	2	3805	idx11_Y09_73	F_2	Female	Yellowish	3
						4019	idx11_Y10_74M	F_2	Male	Yellowish	2	3808	idx11_Y10_74	F_2	Female	Wild type	3
						4023	idx11_Y13_75M	F_2	Male	Yellowish	2	3811	idx11_Y13_75	F_2	Female	Wild type	3
						4024	idx11_Y17_76M	F_2	Male	Wild type	2	3814	idx11_Y17_76	F_2	Female	Yellowish	3
						4025	idx11_Y18_77M	F_2	Male	Wild type	2	3815	idx11_Y18_77	F_2	Female	Wild type	3
						4031	idx11_Y19_78M	F_2	Male	Wild type	2	3818	idx11_Y19_78	F_2	Female	Wild type	3
						4032	idx11_Y28_79M	F_2	Male	Wild type	2	3820	idx11_Y28_79	F_2	Female	Wild type	3
						4033	idx11_Y29_80M	F_2	Male	Wild type	2	3821	idx11_Y29_80	F_2	Female	Wild type	3
						4037*	idx12_Y01_81M	F_2	Male	Wild type	2	3823	idx12_Y01_81	F_2	Female	Wild type	3
						4038*	idx12_Y02_82M	F_2	Male	Wild type	2	4012	idx12_Y02_82	F_2	Female	Wild type	3
						4168	idx12_Y03_83M	F_2	Male	Yellowish	2	4013	idx12_Y03_83	F_2	Female	Wild type	3
						4169	idx12_Y04_84M	F_2	Male	Wild type	2	4018	idx12_Y04_84	F_2	Female	Wild type	3
						4170	idx12_Y05_85M	F_2	Male	Wild type	2	4022	idx12_Y05_85	F_2	Female	Wild type	3
						4171	idx12_Y06_86M	F_2	Male	Wild type	2	4027	idx12_Y06_86	F_2	Female	Wild type	3
						4173	idx12_Y07_87M	F_2	Male	Yellowish	2	4028	idx12_Y07_87	F_2	Female	Yellowish	3
						4174	idx12_Y08_88M	F_2	Male	Wild type	2	4030	idx12_Y08_88	F_2	Female	Wild type	3
						4175	idx12_Y09_89M	F_2	Male	Wild type	2	4034	idx12_Y09_89	F_2	Female	Wild type	3
						4176	idx12_Y10_90M	F_2	Male	Wild type	2	4036	idx12_Y10_90	F_2	Female	Yellowish	3
						4179	idx12_Y13_91M	F_2	Male	Yellowish	2	4039*	idx12_Y13_91	F_2	Female	Wild type	3
						4180	idx12_Y17_92M	F_2	Male	Yellowish	2	4040	idx12_Y17_92	F_2	Female	Wild type	3
						4181	idx12_Y18_93M	F_2	Male	Wild type	2	4041	idx12_Y18_93	F_2	Female	Wild type	3
						4182	idx12_Y19_94M	F_2	Male	Wild type	2	4172	idx12_Y19_94	F_2	Female	Wild type	3
						4185	idx12_Y28_95M	F_2	Male	Wild type	2	4177*	idx12_Y28_95	F_2	Female	Wild type	3
						9999	idx12_Y29_96M	F_2	Male	Yellowish	2	4183	idx12_Y29_96	F_2	Female	Wild type	3

*Samples that are not used for construction of the genetic map and in a case-control association test.

Table S6. Sequences of P1 and P2 adaptors.

P1 adaptor	Nucleotide sequence
GCATG_EcoRI_P1.1	5' ACACTCTTTCCCTACACGACGCTCTTCCGATCTGCATG 3'
AACCA_EcoRI_P1.1	5' ACACTCTTTCCCTACACGACGCTCTTCCGATCTAACCA 3'
CGATC_EcoRI_P1.1	5' ACACTCTTTCCCTACACGACGCTCTTCCGATCTCGATC 3'
TCGAT_EcoRI_P1.1	5' ACACTCTTTCCCTACACGACGCTCTTCCGATCTTCGAT 3'
TGCAT_EcoRI_P1.1	5' ACACTCTTTCCCTACACGACGCTCTTCCGATCTTGCAT 3'
CAACC_EcoRI_P1.1	5' ACACTCTTTCCCTACACGACGCTCTTCCGATCTCAACC 3'
GGTTG_EcoRI_P1.1	5' ACACTCTTTCCCTACACGACGCTCTTCCGATCTGGTTG 3'
AAGGA_EcoRI_P1.1	5' ACACTCTTTCCCTACACGACGCTCTTCCGATCTAAGGA 3'
AGCTA_EcoRI_P1.1	5' ACACTCTTTCCCTACACGACGCTCTTCCGATCTAGCTA 3'
ACACA_EcoRI_P1.1	5' ACACTCTTTCCCTACACGACGCTCTTCCGATCTACACA 3'
ACTGG_EcoRI_P1.1	5' ACACTCTTTCCCTACACGACGCTCTTCCGATCTACTGG 3'
ATTAC_EcoRI_P1.1	5' ACACTCTTTCCCTACACGACGCTCTTCCGATCTATTAC 3'
CATAT_EcoRI_P1.1	5' ACACTCTTTCCCTACACGACGCTCTTCCGATCTCATAT 3'
CGAAT_EcoRI_P1.1	5' ACACTCTTTCCCTACACGACGCTCTTCCGATCTCGAAT 3'
GACAC_EcoRI_P1.1	5' ACACTCTTTCCCTACACGACGCTCTTCCGATCTGACAC 3'
GAGAT_EcoRI_P1.1	5' ACACTCTTTCCCTACACGACGCTCTTCCGATCTGAGAT 3'
GCATG_EcoRI_P1.2	5' Phos-AATTCATGCAGATCGGAAGAGCGTCGTGTAGGGAAAGAGTGT 3'
AACCA_EcoRI_P1.2	5' Phos-AATTTGGTTAGATCGGAAGAGCGTCGTGTAGGGAAAGAGTGT 3'
CGATC_EcoRI_P1.2	5' Phos-AATTGATCGAGATCGGAAGAGCGTCGTGTAGGGAAAGAGTGT 3'
TCGAT_EcoRI_P1.2	5' Phos-AATTATCGAAGATCGGAAGAGCGTCGTGTAGGGAAAGAGTGT 3'
TGCAT_EcoRI_P1.2	5' Phos-AATTATGCAAGATCGGAAGAGCGTCGTGTAGGGAAAGAGTGT 3'
CAACC_EcoRI_P1.2	5' Phos-AATTGGTTGAGATCGGAAGAGCGTCGTGTAGGGAAAGAGTGT 3'
GGTTG_EcoRI_P1.2	5' Phos-AATTCAACCAGATCGGAAGAGCGTCGTGTAGGGAAAGAGTGT 3'
AAGGA_EcoRI_P1.2	5' Phos-AATTTCCTTAGATCGGAAGAGCGTCGTGTAGGGAAAGAGTGT 3'
AGCTA_EcoRI_P1.2	5' Phos-AATTTAGCTAGATCGGAAGAGCGTCGTGTAGGGAAAGAGTGT 3'
ACACA_EcoRI_P1.2	5' Phos-AATTTGTGTAGATCGGAAGAGCGTCGTGTAGGGAAAGAGTGT 3'
ACTGG_EcoRI_P1.2	5' Phos-AATTCCAGTAGATCGGAAGAGCGTCGTGTAGGGAAAGAGTGT 3'
ATTAC_EcoRI_P1.2	5' Phos-AATTGTAATAGATCGGAAGAGCGTCGTGTAGGGAAAGAGTGT 3'
CATAT_EcoRI_P1.2	5' Phos-AATTATATGAGATCGGAAGAGCGTCGTGTAGGGAAAGAGTGT 3'
CGAAT_EcoRI_P1.2	5' Phos-AATTATTCGAGATCGGAAGAGCGTCGTGTAGGGAAAGAGTGT 3'
GACAC_EcoRI_P1.2	5' Phos-AATTGTGTCAGATCGGAAGAGCGTCGTGTAGGGAAAGAGTGT 3'
GAGAT_EcoRI_P1.2	5' Phos-AATTATCTCAGATCGGAAGAGCGTCGTGTAGGGAAAGAGTGT 3'

P2 adaptor	Sequence
MseI_P2.1	5' GTGACTGGAGTTCAGACGTGTGCTCTTCCGATCT 3'
MseI_P2.2	5' Phos-TAAGATCGGAAGAGCGAGAACAA 3'

Phos, phosphorylated.

Table S7. Sequences of PCR primers for ddRAD-seq.

Name	Nucleotide sequence
PCR1	5' AATGATACGGCGACCACCGAGATCTACACTCTTTCCCTACACGACG 3'
PCR2_Idx_1_ATCACG	5' CAAGCAGAAGACGGCATACGAGATCGTGATGTGACTGGAGTTCAGACGTGTGC 3'
PCR2_Idx_5_ACAGTG	5' CAAGCAGAAGACGGCATACGAGATCACTGTGTGACTGGAGTTCAGACGTGTGC 3'
PCR2_Idx_6_GCCAAT	5' CAAGCAGAAGACGGCATACGAGATATTGGCGTGACTGGAGTTCAGACGTGTGC 3'
PCR2_Idx_8_ACTTGA	5' CAAGCAGAAGACGGCATACGAGATTCAAGTGTGACTGGAGTTCAGACGTGTGC 3
PCR2_Idx_11_GGCTAC	5' CAAGCAGAAGACGGCATACGAGATGTAGCCGTGACTGGAGTTCAGACGTGTGC 3
PCR2_Idx_12_CTTGTA	5' CAAGCAGAAGACGGCATACGAGATTACAAGGTGACTGGAGTTCAGACGTGTGC 3

PCR1 contains a sequence complimentary to P1 adaptor sequences and the others to P2 adaptor sequences. The last six chacacters in the names of PCR2 primers show nucleotide sequences of PCR indexs.

Table S8. Sequences of primers used for direct sequencing, PCR-RFLP, and real time PCR.

Name of primer or primer pair	Name of oligonucleotide	Purpose	Nucleotide sequence	Length (nt)	Tm	Ta	Product length (bp)
pmel_1	pmel_1_24Fw	Amplification of cDNA and direct sequencing	5' CTTTGCCTCTCTCGGAGTCGGAG 3	23	63.1	60	1,369
	pmel_1_1368Rv		5' CATCCACTTCCACATCTGCAGCA 3	24	61.3		
pmel_2	pmel_2_1024Fw	Amplification of cDNA and direct sequencing	5' CTCCTTGGGACCTACAGCTACAC 3	23	61.3	60	1,472
	pmel_2_2353Rv		5' AGTCTTTATTAGCAGCCAGTACAC	25	56.3		
—	pmel-seq_176Rv	Direct sequencing	5' CCGTCCCCTCCTTCCACG 3'	18	61.7	—	—
—	pmel_3_1683Fw	Direct sequencing	5' CTGTATCGCTATGGCTCCTTCTC 3'	23	59.6	—	—
pmel_RFLP	pmel_RFLP-F	PCR-RFLP	5' CAAAGCCACCTTCTCCATCTCTC 3'	23	60.9	55	445
	pmel_RFLP-R		5' CATCCGTCCCTACTGTCAGCTTC 3'	23	62.6		
pmel_mRNA_RT-PCR5	pmel_mRNA_RT-PCR5_Fw	Real time PCR	5' ATCAGCAACGACGCTCCCA 3'	19	57.3	60	139
	pmel_mRNA_RT-PCR5_Rv		5' CCATGTGGGTGCCGTTAACG 3'	20	59.5		
chick-actb	chick-actb_Fw	Real time PCR	5' GCTACAGCTTCACCACCACA 3'	23	59.6	60	90
	chick-actb_Rv		5' TCTCCTGCTCGAAATCCAGT 3'	23	57.8		

Tm, melting temprature (°C); Ta, annealing temprature (°C).

Thermocycling for amplification of *PMEL* cDNA: 2 min at 94°C and 45 cycles of (10 sec at 98°C, 30 sec at 60°C and 30 sec/kb at 68°C).

Thermocycling for amplification for PCR-RFLP: 5 min at 95°C and 40 cycles of (30 sec at 95°C, 30 sec at 55°C, and 60 sec at 72°C).

Thermocycling for real time PCR:10 min at 95°C, 40 cycles of (15 sec at 95°C and 1 min at 60°C), 15 sec at 95°C, 1 min at 60°C, and 15 sec at 95°C.

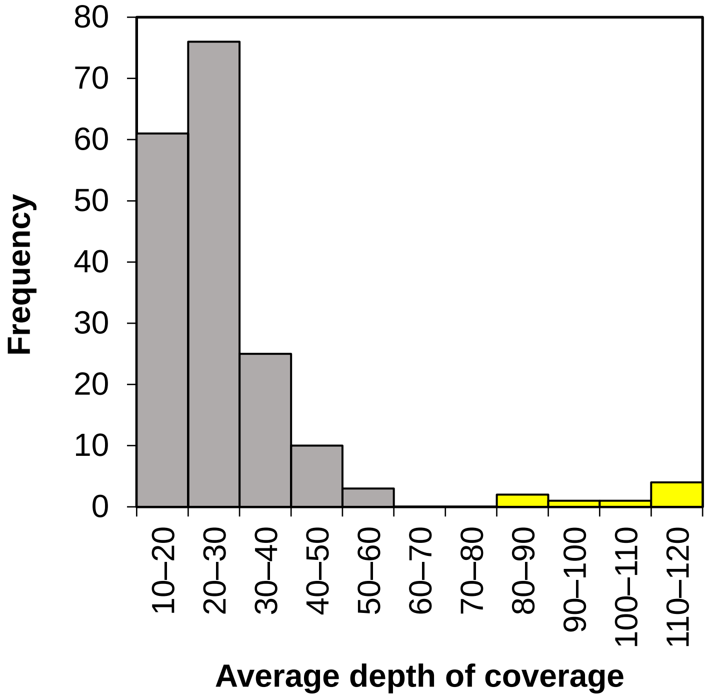

Figure S1. Distribution of average coverage depth of SNP markers. Yellow bars represent two parent samples that were analysed in both the first and second sequencing, and six samples of F_2 offspring that were analysed in the first sequencing. Grey bars represent 190 samples of F_2 offspring that were analysed in either the second or third sequencing. The median of the average coverage depth is 108.8× in samples represented by yellow bars and 23.1× in samples represented by grey bars.

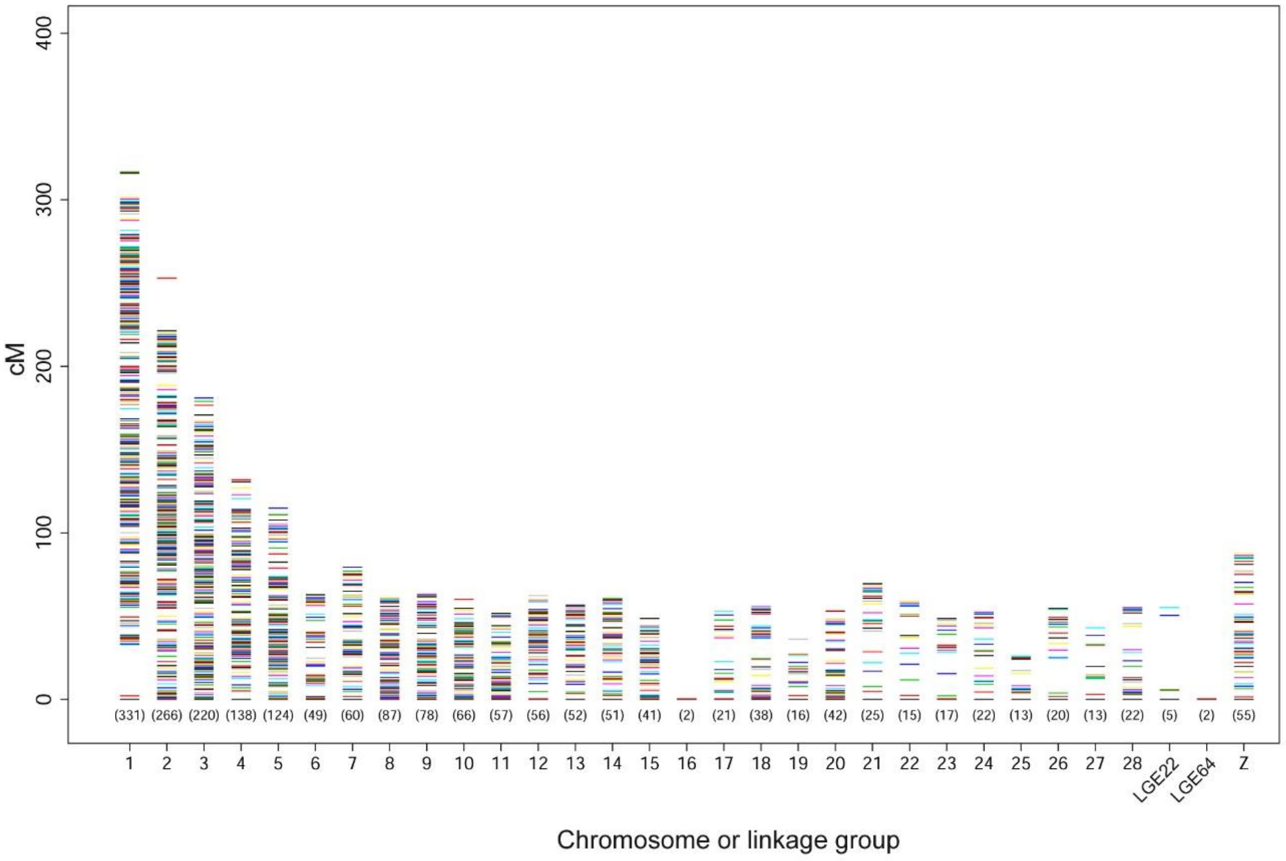

Figure S2. Genetic map constructed with 2,004 SNP markers. The horizontal axis represents chromosomes or linkage groups. The vertical axis represents genetic distance (cM). Each coloured line represents a SNP marker. Numerical values in parenthesis at the bottom indicate the total number of SNP markers in each chromosome or linkage group.

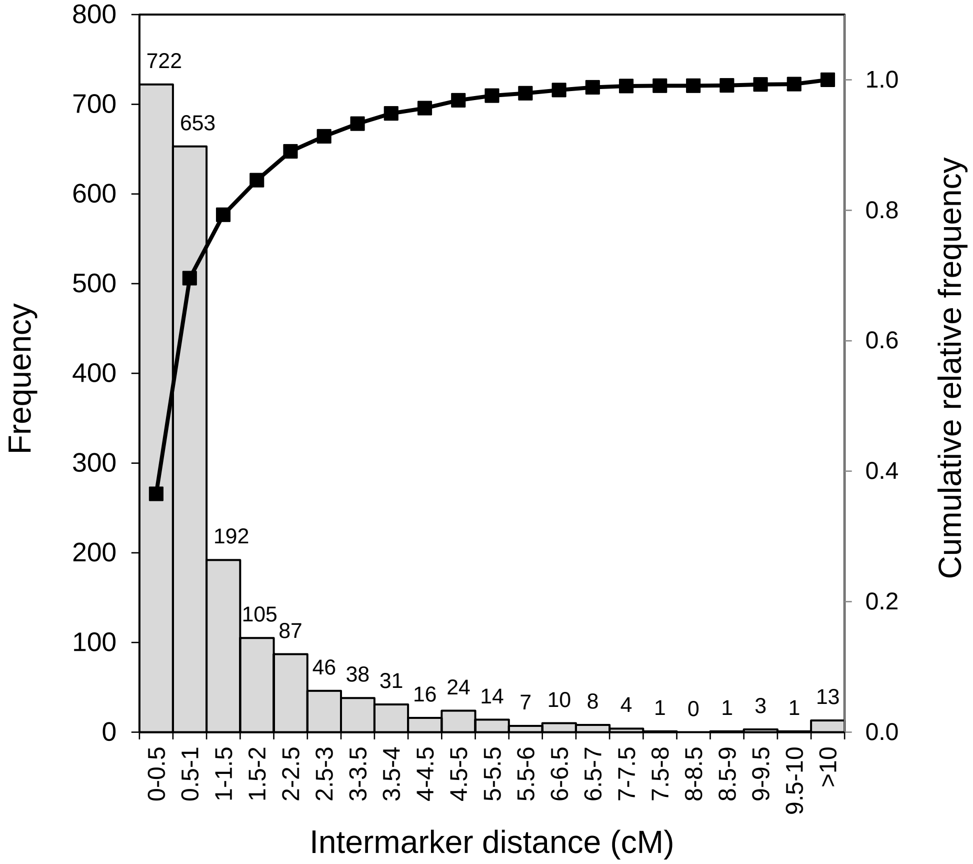

Figure S3. Distribution of intermarker distance in the genetic map. Histogram shows the frequency of each intermarker distance. Dots show the cumulative relative frequency of each intermarker distance.

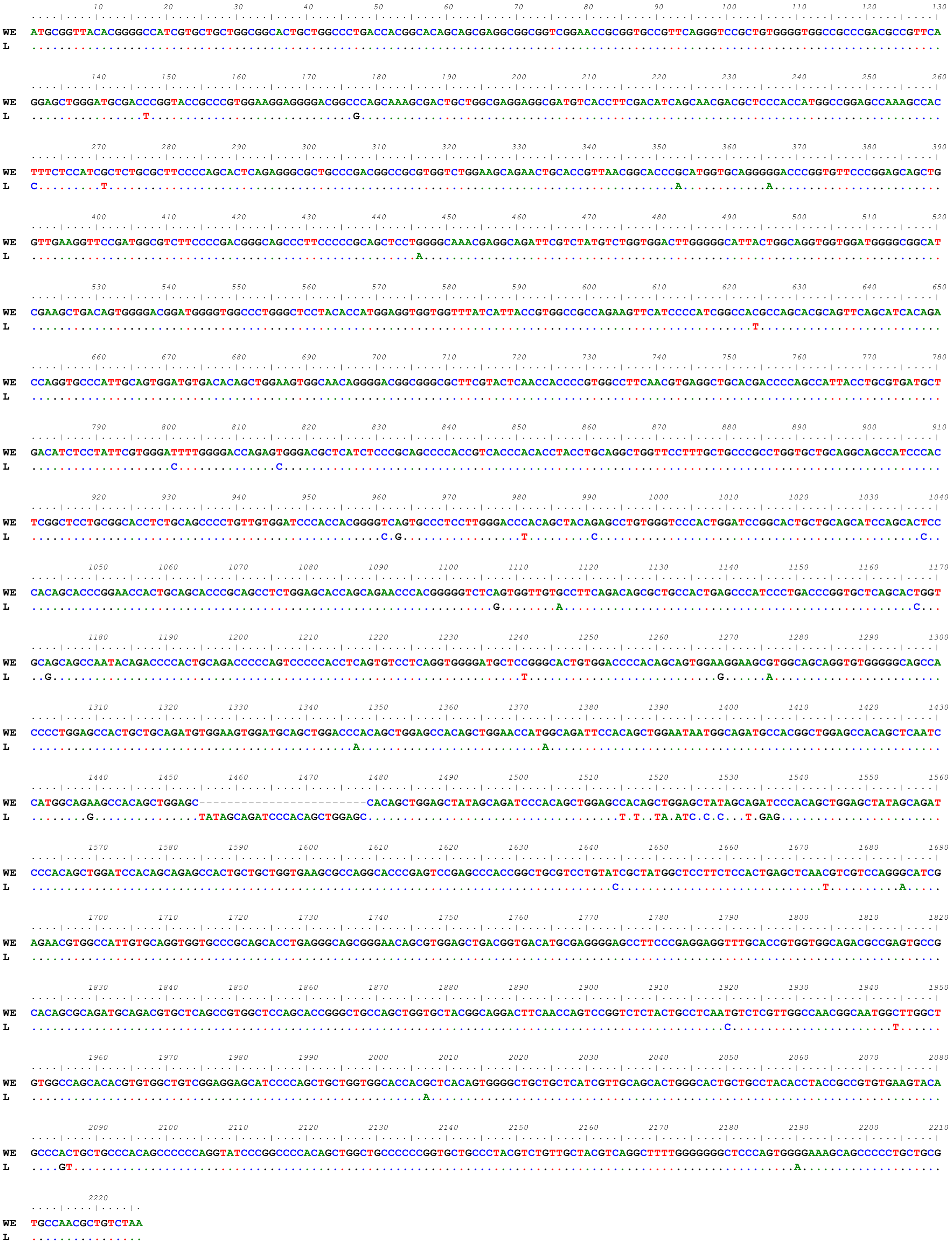

Figure S4. *PMEL* coding sequences of the WE and L strains. A total of 47 base substitutions, including G>A at the 446th nucleotide, and a single 24-bp insertion/deletion at the 1455–1478th nucleotides are present.

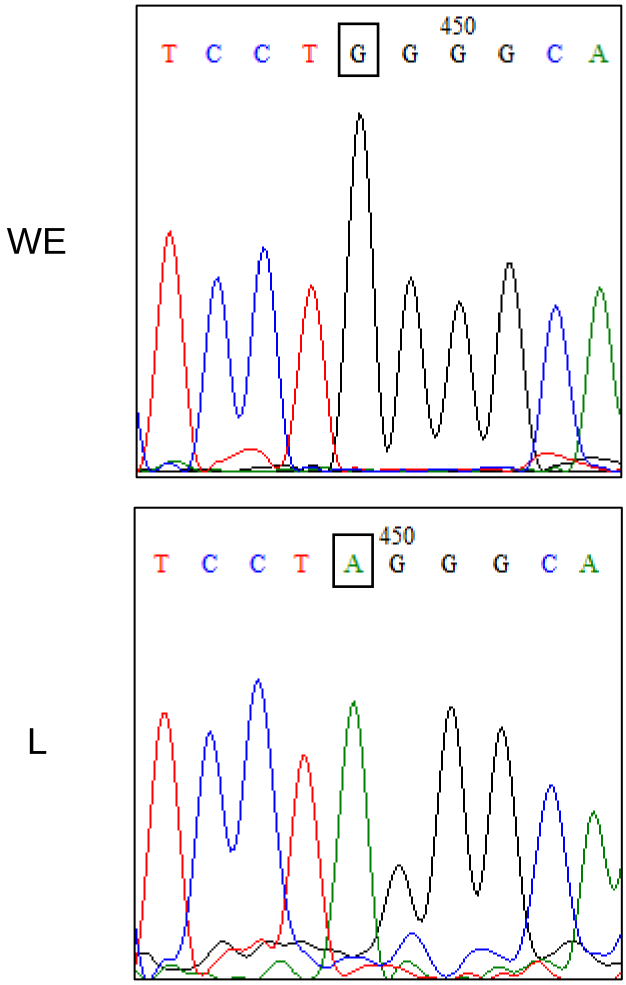

Figure S5. ABI chromatograms of nucleotide sequences around the 446th nucleotide (squared frame) in the coding sequence of *PMEL*. The 446th nucleotide is guanine in the WE strain and adenine in the L strain.

```
                10        20        30        40        50        60
      ....|....|....|....|....|....|....|....|....|....|....|....|
WE    MRLHGAIVLLAALLALTTAQQRGGGRNRGAVQGPLWGGRPTPFRSWDATRYRPWKEGTAQ
L     ............................................................

                70        80        90       100       110       120
      ....|....|....|....|....|....|....|....|....|....|....|....|
WE    QSDCWRGGDVTFDISNDAPTMAGAKATFSIALRFPSTQRALPDGRVVWKQNCTVNGTRMV
L     ..............................S..........................H..

               130       140       150       160       170       180
      ....|....|....|....|....|....|....|....|....|....|....|....|
WE    QGDPVFPEQLVEGSDGVFPDGQPFPRSSWGKRGRFVYVWWTWGHYWQVVDGAASKLTVGT
L     ............................*

               190       200       210       220       230       240
      ....|....|....|....|....|....|....|....|....|....|....|....|
WE    DGVALGSYTMEVVVYHYRGRQKFIPIGHASTQFSITDQVPIAVDVTQLEVATGDGGRFVL
L

               250       260       270       280       290       300
      ....|....|....|....|....|....|....|....|....|....|....|....|
WE    NHPVAFNVRLHDPSHYLRDADISYSWDFGDQSGTLISRSPTVTHTYLQAGSFAARLVLQA
L

               310       320       330       340       350       360
      ....|....|....|....|....|....|....|....|....|....|....|....|
WE    AIPLGSCGTSAAPVVDPTTGSVPSLGPTATEPVGPTGSGTAAASSTPTAPGTTAAPAASG
L

               370       380       390       400       410       420
      ....|....|....|....|....|....|....|....|....|....|....|....|
WE    APAEPTGVSVVVPSDSAATEPIPDPVLSTGAAANTDPTADPQSPTSVSSGGDAPGTVDPT
L

               430       440       450       460       470       480
      ....|....|....|....|....|....|....|....|....|....|....|....|
WE    AVEGSVAAGVGAATPGATAADVEVDAAGPTAGATAGTMADSTAGIMADATAGATAQSMAE
L

               490       500       510       520       530       540
      ....|....|....|....|....|....|....|....|....|....|....|....|
WE    ATAGATAGAIADPTAGATAGAIADPTAGAIADPTAGSTAEPLLLVKRQAPESEPTGCVLY
L

               550       560       570       580       590       600
      ....|....|....|....|....|....|....|....|....|....|....|....|
WE    RYGSFSTELNVVQGIENVAIVQVVPAAPEGSGNSVELTVTCEGSLPEEVCTVVADAECRT
L

               610       620       630       640       650       660
      ....|....|....|....|....|....|....|....|....|....|....|....|
WE    AQMQTCSAVAPAPGCQLVLRQDFNQSGLYCLNVSLANGNGLAVASTRVAVGGASPAAGGT
L

               670       680       690       700       710       720
      ....|....|....|....|....|....|....|....|....|....|....|....|
WE    TLTVGLLLIVAALGTAAYTYRRVKYSPLLPTAPQVSRPHSWLPPGAALRLLLRQAFGGAP
L

               730
      ....|....|....
WE    SGESSPLLRANAV*
L
```

Figure S6. Amino acid sequences of PMEL in the WE and L strains. A premature stop codon is present at the position of the 149th amino acid in the L strain. The truncated form of PMEL contains two amino acid substitutions, Ala91Ser and Arg118His.

```
                            Signal sequence
Quail (WE)   1   --MRLHGAIV LLAALLALTT AQQRGGGRNR GAVQGPLWGG RPTPFRSWDA TRYRPWKEGT  58
Chicken      1   --MRLHGAIV LLAALLALVT AQQRGGGRSR GGVKGSAWGG RPAPFRSWDT ARYRPWQEGT  58
Human        1   MDLVLKRCLL HLAVIGALLA VGATKVPRNQ DWLG----VS RQLRTKAWNR QLYPEWTE--  54

Quail (WE)   59  AQQSDCWRGG DVTFDISNDA PTMAGAKATF SIALRFPSTQ RALPDGRVVW KQNCTVNGTR  118
Chicken      59  ARQNDCWRGG DVTFDISNDA PTLVGARATF SIALRFPGTQ TVLPDGRVVW SQNCTVNGTR  118
Human        55  AQRLDCWRGG QVSLKVSNDG PTLIGANASF SIALNFPGSQ KVLPDGQVIW VNNTIINGSQ  114

Quail (WE)   119 MVQGDPVFPE QLVEGSDGVF PDGQPFPRSS WGKRGRFVYV WWTWGHYWQV VDGAASKLTV  178
Chicken      119 MLQGDPVYPE QLAEGSDGVF PDGQPFPRSA WGKRGRFVYV WWTWGRYWQV VDGATSQLTV  178
Human        115 VWGGQPVYPQ ETDDAC--IF PDGGPCPSGS WSQKRSFVYV WKTWGQYWQV LGGPVSGLSI  172

Quail (WE)   179 GTDGVALGSY TMEVVVYHYR GRQKFIPIGH ASTQFSITDQ VPIAVDVTQL EVATGDGGRF  238
Chicken      179 GTDGVALGSY TMEVVVYHYR GRQRFIPIGH ASTQFSITDQ VPIAVDVTQL EVAAGDGGSF  238
Human        173 GTGRAMLGTH TMEVTVYHRR GSRSYVPLAH SSSAFTITDQ VPFSVSVSQL RALDGGNKHF  232
                                                    PKD domain
Quail (WE)   239 VLNHPVAFNV RLHDPSHYLR DADISYSWDF GDQSGTLISR SPTVTHTYLQ AGSFAARLVL  298
Chicken      239 VRNRPVAFNV RLHDPSHYLR DADISYSWDF GDQSGTLISR SPTVTHTYLQ AGSFAARLVL  298
Human        233 LRNQPLTFAL QLHDPSGYLA EADLSYTWDF GDSSGTLISR ALVVTHTYLE PGPVTAQVVL  292

Quail (WE)   299 QAAIPLGSCG TSAAPVVDPT TGSVPSLGPT ATEPVGPTGS GTAAASS--- ---TPTAPGT  352
Chicken      299 QAAIPLSSCG TSAPPVVDPT TGPVPSLGPT ATQPVGPTGS GTATAPSNLT GSGTAAAPGT  358
Human        293 QAAIPLTSCG SS------PV PGTTDGHRPT AEAPN----- TTAGQVP--- ---TTEVVGT  335

Quail (WE)   353 TAAPAASGAP AEPTGVSVVV PSDSAATEPI PDPVLSTG-- -AAANTDPTA DPQSPTSVSS  409
Chicken      359 TAAPRASGAP AEPTGVSVAV LSDSAATEPL PDPVLSTAVA DAAAGTDPTA DPLPPTSVSS  418
Human        336 TPGQAPTAEP SGTTSVQVPT ------TEVI S--------- --------TA PVQMPTAEST  372

Quail (WE)   410 GGDAPGTVDP TAVEGSVAAG VG------AA TPGATAADVE VDAAG----- ---PTAGATA  455
Chicken      419 GGDAPGTVAP TAVEGSVAAG VGTAEDVAAA TPGATAADVA VDTAGATDGD AVGPTAAATA  478
Human        373 G-MTPEKVPV SEVMGTTLAE MS-----TPE ATGMTPAEVS IVVLS----- ----------  411
                           Repeat 1                          Repeat 2
Quail (WE)   456 GTMADSTAGI MADATAGATA QSMAEATAGA TAGAIADPTA GATAGAIADP TAGAIADP--  513
Chicken      479 ESIADPTAGA TDGDAVGATA ESIADPTAGA TDGDAVGPTA AATAESIADP TAGATAVSSG  538
Human        411 ---GTTAAQV TTTEWVETTA RELPIPEPEG PDASSIMSTE SITG------ SLGPLLDG--  460
                       Proteolytic cleavage site
Quail (WE)   513 --TAGSTAEP LLLVKRQAPE SEPTGCVLYR YGSFSTELNV VQGIENVAIV QVVPAAPEGS  571
Chicken      539 SATAGATAEP LLLVKRQAPE AEPTGCVLYR YGTFSTELNI VQGIESVAIV QVVPAAPEGS  598
Human        460 -------TAT LRLVKRQVP- ---LDCVLYR YGSFSVTLDI VQGIESAEIL QAVPS---GE  506

Quail (WE)   572 GNSVELTVTC EGSLPEEVCT VVADAECRTA QMQTCSAVAP APGCQLVLRQ DFNQ-SGLYC  630
Chicken      599 GNSVELTVTC EGSLPEEVCT VVADAECRTA QMQTCSAVAP APGCQLVLRQ DFNQ-SGLYC  657
Human        507 GDAFELTVSC QGGLPKEACM EISSPGCQPP AQRLCQPVLP SPACQLVLHQ ILKGGSGTYC  566
                                                  Transmembrane domain
Quail (WE)   631 LNVSLANGNG LAVASTRVAV GGASPAAGGT TLTVGLL--- LIVAALGTAA YTYRRVKYSP  687
Chicken      658 LNVSLANGNG LAVASTHVAV GGASPAASGT TLTVGLLWAP LMAAALGTAA YTYRRVKYSP  717
Human        567 LNVSLADTNS LAVVSTQLIM PGQEAGLGQV PLIVGIL--- LVLMAVVLAS LIYRRR----  619
                          Cytoplasmic domain
Quail (WE)   688 LLPTAPQVSR PHSWLPPGAA LRLLLRQAFG GAPSGESSPL LRANAV 733
Chicken      718 LLPTAPTAPR PHSWLPPGAT LRLLLRQAFG GAPSGESSPL LRANAV 763
Human        620 LMKQDFSVPQ ----LPHSSS HWLRLPRIFC SCPIGENSPL LSGQQV 661
```

Figure S7. Predicted protein domains of quail PMEL. Amino acid sequences of PMEL in the WE strain of quail, chicken (NP_990443.2), and human (NP_001186983.1) are aligned. Protein motifs, except for the polycystic kidney disease (PKD) domain, were predicted based on amino acid sequence homology between quail and chicken PMEL (Kerje et al. 2004). The PKD domain was predicted using Pfam 31.0 (http://pfam.xfam.org/) [The Pfam protein families database: towards a more sustainable future (doi: 10.1093/nar/gkv1344)].

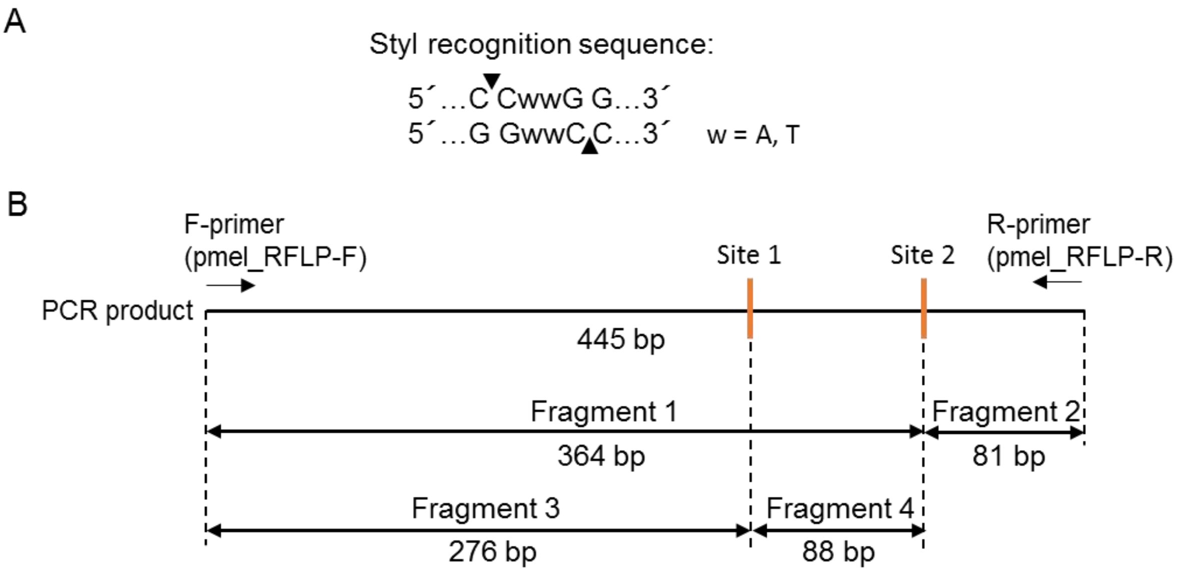

	Site 1	Site 2
Father (WE)	C CTGG G	C CATG G
Mother (L)	C CTAG G	C CATG G

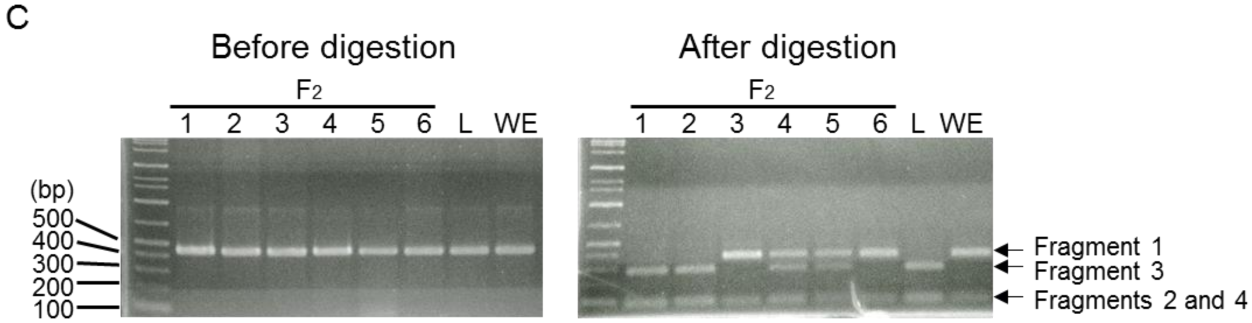

Figure S8. Schematic representation of PCR-RFLP analysis to determine genotypes at the site of the candidate mutation. (**A**) Schematic diagram of StyI recognition sequence. (**B**) Schematic diagram of PCR product and restriction fragments. Site 1 is a 6-bp sequence that contains the site of the candidate mutation for the *yw* phenotype, which is shown by red-colored G and A in the table. Site 1 is recognized by StyI in the maternal genome, but not in the paternal genome. Site 2 is recognized by StyI in both parents. Restriction enzyme digestion of a 445-bp PCR product yields two DNA fragments (fragments 1 and 2) from the paternal DNA, three DNA fragments (fragments 2–4) from the maternal DNA, and four DNA fragments (fragments 1–4) from heterozygotes. (**C**) Representative result of PCR-RFLP analysis, which indicates genotypes of six F_2 individuals (F_2) and parents (L and WE) at the site of the candidate mutation. The left image shows banding patterns of PCR products before digestion and the right image shows those after digestion. The original image for these gel images is shown in Fig. S10.

A

	Strain	Plumage color		Strain	Plumage color
1	L	Yellowish	6	rb-TKP	Black (recessive)
2	WE	Normal	7	French	Yellow (dominant)
3	AMRP	Panda (normal color spotting, recessive)	8	W (Wild)	Normal
4	Quv	Normal	9	JW	Normal
5	RWN	Normal	10	Estonian	Panda

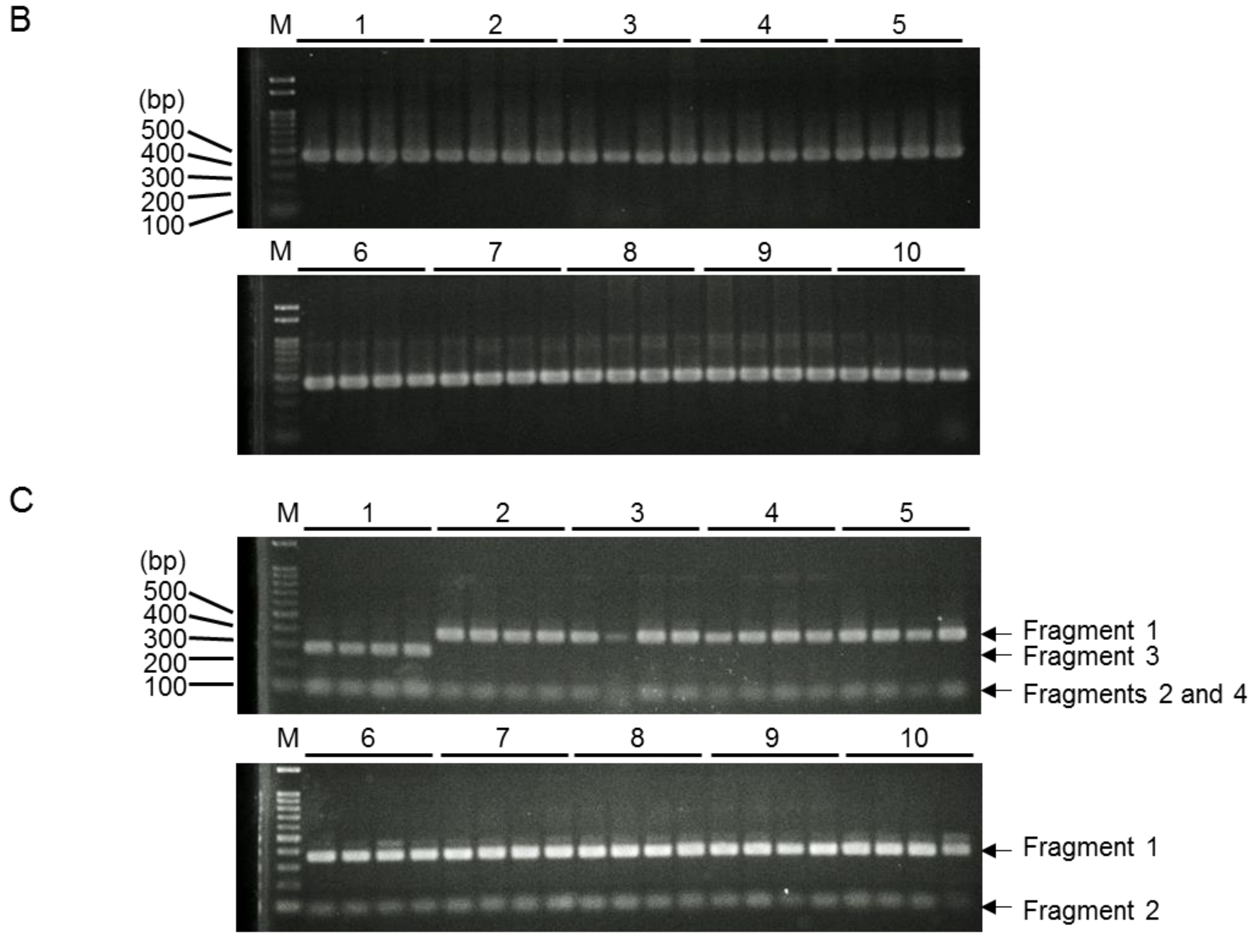

Figure S9. PCR-RFLP analysis for detection of the candidate mutation in ten quail strains. (**A**) Ten quail strains were used for analysis. (**B, C**) RFLP analysis of PCR products by agarose gel electrophoresis. PCR products before digestion (**B**) and those after digestion with StyI (**C**). ‘M’ indicates the lanes for the DNA ladder marker. Numbers above the images indicate the strains shown in (**A**). Four individuals were used for each strain. All individuals of the L strain were homozygous for the mutant allele; however, the others do not show that genotype. Original images for these gel images are shown in Fig. S10.

A

B

C

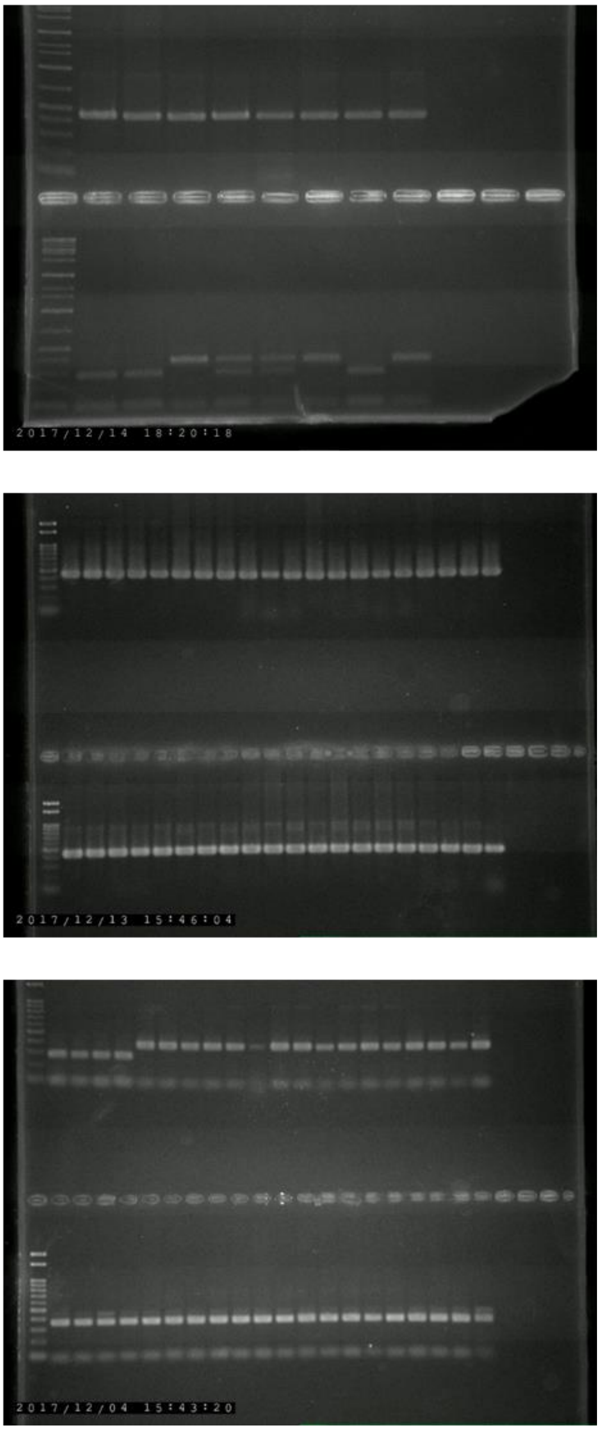

Figure S10. Images of agarose gels used for Figs. S8 and S9 (**A**) Image of a gel used for Fig. S8. The upper part shows banding patterns of PCR products before digestion and the lower part shows those after digestion. (**B, C**) Images of gels used for Figs. S9B and C show banding patterns of PCR products before digestion (**B**) and those after digestion (**C**).
